# Visual features explain dynamic aesthetic experiences across distinct movie content

**DOI:** 10.1038/s44271-026-00531-7

**Published:** 2026-09-11

**Authors:** Mustafa Alperen Ekinci, Nina Buhlmann, Daniel Kaiser

**Affiliations:** 1 Neural Computation Group, Justus Liebig University Giessen, Giessen, Germany; 2 Center for Mind, Brain and Behavior (CMBB), Universities of Giessen, Marburg and Darmstadt, Marburg, Germany; 3 Center for Applied Computer Science and Data Science (ZAD), Justus Liebig University Giessen, Giessen, Germany; 4 Cluster of Excellence “The Adaptive Mind”, Universities of Giessen, Marburg and Darmstadt, Giessen, Germany

**Keywords:** Perception, Human behaviour

## Abstract

Aesthetic experiences in everyday life unfold under continuously changing visual input. Although these experiences clearly depend on the observer and context, they are partly explained by the visual features of the input. Here, we investigated how well a combination of visual features predicts dynamic aesthetic experiences during naturalistic and artistic movie watching. In two experiments, participants continuously rated the aesthetic appeal of either the nature documentary *Home* (N = 37) or the animated art-style movie *Loving Vincent* (N = 30). We modeled moment-to-moment ratings using image-computable visual features extracted from each movie frame, including visual fluency, color and motion statistics, and symmetry. Linear models trained on these features reliably predicted aesthetic ratings for new movie parts, both within and across observers, pointing to shared perceptual influences on aesthetic experiences. Model comparisons showed that visual fluency and color-related features were most informative for predicting aesthetic experience in both movies. Critically, models trained on one movie could reliably predict aesthetic appeal ratings in the other movie, despite the movies’ remarkably different content and styles. Color features were most informative for cross-movie prediction. We conclude that visual features provide a robust basis for explaining dynamic aesthetic experiences across observers and different movie content.

## Introduction

Complex and dynamic visual stimuli are often associated with aesthetic experiences. In cinema, certain movie scenes captivate us with their beauty, such as the breathtaking New Zealand landscapes in *The Lord of the Rings* trilogy, whereas others remain visually unremarkable. This observation extends beyond cinema: hiking through those same New Zealand landscapes should be equally beautiful, while our everyday walk to work is often less so.

What factors drive these differences in aesthetic appeal? Research on visual aesthetics has linked aesthetic experiences to sensory and perceptual processing: some visual features are reliably connected to perceiving beauty, while others predispose reduced beauty^1–8^. For example, people tend to prefer curved over sharp-edged objects^9^, and symmetric over asymmetric faces^10^. While we have learned a lot about which features are connected to the perception of beauty, the current literature remains highly fragmented on two grounds: prior work has often focused on (i) individual features in isolation^11,12^ and (ii) just one type of visual content^6,13,14^.

First, aesthetic preferences have largely been examined with respect to one candidate visual feature at a time^15^. Such studies showed that preferences are influenced by low-level properties like luminance, contrast, and color^16–18^, as well as structural mid- and higher-level features like symmetry, complexity, or curvature^19–21^. On a configural level, Gestalt principles^22^ and visual fluency^23,24^ have been connected to perceived beauty. Yet most of these studies only looked at one or relatively few of these features at a time, rendering it unclear how well aesthetic appeal can be modeled once all candidate features are considered.

Second, research on aesthetic preferences has typically examined the importance of visual features within a single type of visual content, such as artworks^14,25,26^, objects^27,28^, faces^29,30^, or natural scenes^31,32^. This domain-specific focus has limited insight into how aesthetic appeal generalizes across different visual content, and whether the inferred importance of certain feature configurations holds true when different types of complex visual stimuli are considered.

As a consequence, we currently know little about how multiple visual properties jointly contribute to aesthetic judgments and how such feature-based accounts generalize across different content. Here, we address these limitations by examining a comprehensive set of visual features across two movies that differ markedly in both their visual style and semantic content. Whereas *Home* is a nature documentary about the Earth and humanity’s relationship with its natural resources, depicting diverse real-world landscapes and environmental scenes with varying levels of aesthetic appeal, *Loving Vincent* is an animated biographical film that retells the life of Vincent van Gogh through a highly stylized painterly aesthetic. Thus, the two movies differ substantially not only in their visual appearance but also in their semantic content, narrative structure, and emotional themes.

**Fig. 1:**
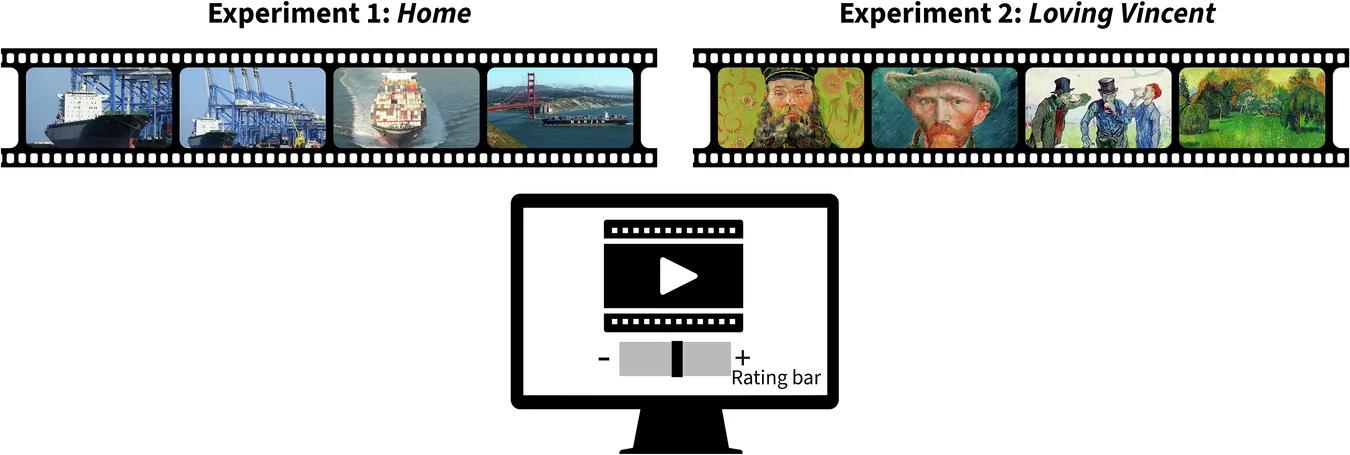
Experiment paradigm. Participants continuously rated the aesthetic appeal of the movies *Home* (Experiment 1) or *Loving Vincent* (Experiment 2) using a slider bar. Images shown are illustrative and were not used as experimental stimuli. The original experimental stimuli were the movies *Home* and* Loving Vincent*. Images are licensed under CC BY 4.0. Photographers, in order: Sludge G, Daniel Ramirez, John Morgen, Free Public Domain Illustrations. Images were obtained through Openverse.

**Fig. 2:**
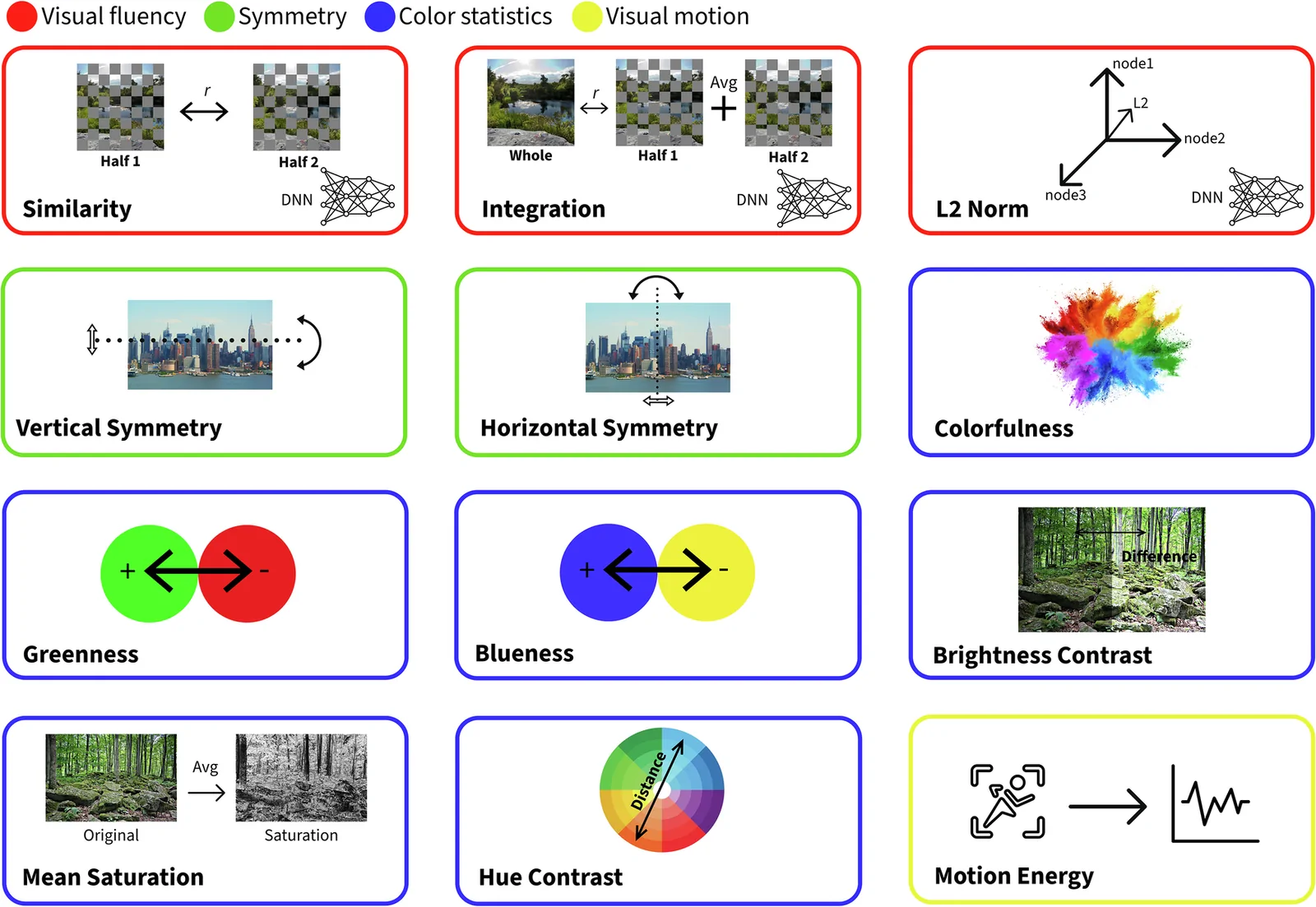
Image-computable predictors. We extracted twelve visual predictor metrics from individual movie frames. Predictors comprised four groups, covering aspects of visual fluency (similarity, integration, and L2 norm), symmetry (vertical and horizontal symmetry), color statistics (colorfulness, greenness, blueness, mean saturation, brightness contrast, and hue contrast), and visual motion (motion energy). Images shown are illustrative and were not used as experimental stimuli. The original experimental stimuli were the movies *Home* and * Loving Vincent*. Images are licensed under CC BY 4.0. Photographers, in order: Ruben Holthuijsen, Targut, Eva Abreu, Dominic Alves. Images were obtained through Openverse.

In two experiments, our participants watched either *Home* (Experiment 1) or *Loving Vincent* (Experiment 2). In both experiments, they continuously rated the movies’ aesthetic appeal on a moment-to-moment level (Fig. 1). By modeling continuous aesthetic ratings from the image-computable features extracted on a frame-to-frame level (Fig. 2), we test whether (i) visual features reliably predict aesthetic experiences in free-flowing movies, (ii) how these predictions generalize across observers, and (iii) how they generalize across the two different movies.

For both movies, we extracted twelve image-computable features grouped into four broad categories: visual fluency, symmetry, color statistics, and motion energy (Fig. 2). These features were selected because previous empirical studies have identified them as important predictors of aesthetic preference. Specifically, visual fluency, symmetry, and color statistics have each been associated with aesthetic judgments across a variety of static visual stimuli, whereas motion energy was included to examine whether dynamic visual information provides an additional contribution to aesthetic experiences during movie viewing. The visual fluency category included three predictors, similarity, integration, and L2 norm, derived from deep neural network representations. These measures have previously been shown to predict aesthetic preferences across multiple datasets of naturalistic images^33^. They capture mid-level spatial organization of an image by quantifying how efficiently different parts of an image are integrated within a whole, allowing us to test whether deep neural network-based measures of visual fluency also explain dynamic aesthetic experiences during movie viewing. Symmetry has been linked to aesthetic preference across a wide range of visual stimuli^34,35^. Accordingly, we included both vertical and horizontal symmetry computations to examine their contribution to dynamic aesthetic experiences^36^. Lastly, we computed a broad range of color statistics to capture different aspects of color perception. Color is a unique visual feature because it conveys both low-level perceptual information and higher-level emotional and semantic associations, making it an important determinant of aesthetic preference^37,38^. For this reason, we included computational color measures that have previously been shown in aesthetics research: colorfulness, greenness, and blueness^39^, as well as hue contrast, brightness contrast, and mean saturation^4,40^.

## Methods

### Participants

The study comprised two experiments. In Experiment 1, a total of 37 participants with normal or corrected-to-normal vision participated (*M*_*age*_ = 26.76, *SD* = 4.28; 21 females and 16 males). In Experiment 2, 30 participants with normal or corrected-to-normal vision took part (*M*_*age*_ = 26.28, *SD* = 6.11; 15 females and 15 males). Participants self-reported their sex during the experiment. Data on gender identity, socioeconomic status, community of descent, and race/ethnicity were not collected. All participants provided written informed consent and were reimbursed at a rate of €10 per hour for their participation. The study was approved by the General Ethics Committee of Justus Liebig University Giessen (approval no. AZ25/22) and was conducted in accordance with the Declaration of Helsinki.

### Preregistration

This study was not preregistered.

### Movie stimulus

In Experiment 1, participants watched the documentary *Home* divided into nine segments (474.3, 534.8, 529.3, 535.2, 520.7, 537.6, 533.3, 523.3, and 357.7 s; mean duration = 505.1 s). *Home* includes a diverse range of visual content, from the dance of a whale to forest fires, capturing both pleasing and unpleasing scenes from all around the world. In Experiment 2, the animated movie *Loving Vincent* was shown in 8 segments (584.3, 527, 604.6, 620.1, 603.5, 565.1, 544.6, and 601.1 seconds; mean duration = 581.3 s). *Loving Vincent* depicts an animated interpretation of Vincent van Gogh’s paintings and life story. Both movies were presented without sound. The segments did not overlap, and each was cut at the end of a scene to preserve coherence within segments. Movie segments were presented in randomized order, and there were 8 unique presentation sequences in Experiment 1 and 18 unique presentation sequences in Experiment 2. The (differing) number of random sequences was related to an error in implementing the randomization. The movies exhibit relatively weak narrative continuity, which is largely lost in the absence of sound. After collecting data from twelve participants in Experiment 1, we slightly improved movie clipping, removing some black screens, which lasted only around 2 min in total (3.38% of the total duration).

### Procedure

In both experiments, participants sat in a darkened room with their heads stabilized by a chin and forehead rest, positioned 68 cm from the screen. Stimuli were displayed on a monitor with a resolution of 1280 × 720 pixels and a refresh rate of 100 Hz. Movie frames were presented sequentially at their native frame rate of 25 frames/s using Psychtoolbox. At this viewing distance, the stimuli subtended 28.7 × 16.4 degrees of visual angle. A practice session of continuous aesthetic rating was shown at the beginning of each experiment. The experiment was programmed using Psychtoolbox version 3.0.19 in MATLAB R2022a (MathWorks, Natick, MA, USA) on a Windows 10 PC. During the movie segments, each participant watched all segments and rated aesthetic appeal using a slider presented below the movie. Participants adjusted the slider using the right or left arrow keys. The position of the rating slider was sampled once per movie frame (25 Hz). Between button presses, the slider position remained constant, resulting in a frame-by-frame record of participants’ continuous ratings. If participants did not provide a response for more than 5 seconds, the slider flickered red and green. In this case, they could press the up arrow to indicate that their rating was still the same. The text shown in the on-screen instruction was as follows:

*“While watching the video, please continuously rate your aesthetic experience using the LEFT and RIGHT ARROW keys. The rating bar will appear at the bottom of the screen. The center of the bar represents a neutral rating. The right side represents higher aesthetic experience, and the left side represents lower aesthetic experience. If you do not respond within 5 seconds, the rating bar will flicker red and green. While flickering, if your aesthetic experience stays stable, you can press the UP ARROW!”*

### Behavioral data processing

For each participant, we computed the average reaction-time delay in the continuous rating task and adjusted their rating time series accordingly. To accomplish this, movie cuts were identified from framewise visual changes. For each pair of consecutive frames, both frames were converted to grayscale, and the mean pixel-intensity difference across all pixels was computed. Frame transitions exceeding predefined positive or negative thresholds were identified as candidate cuts, and consecutive suprathreshold frames were grouped into a single cut region. The first frame of each cut region was used as the temporal reference point for the latency analysis. Thresholds were decided by trying a range of different values and visually inspecting the resulting cuts for both movies. We then measured the average time to the first rating change following each cut and used this latency to temporally shift the rating data, with the shift limited to a maximum of two seconds. The mean estimated latency before applying the two-second cap was 62.1 frames in Experiment 1 (*Home*) and 55.2 frames in Experiment 2 (*Loving Vincent*). As a robustness check, we also evaluated fixed temporal shifts ranging from 0 to 5 s in 0.5-s increments and compared the resulting prediction performance to individually estimated delays (Fig. S6). Furthermore, the first two seconds of each movie segment were excluded, as participants required additional time to adjust their ratings at segment onsets.

### Image-computable predictors

The following image-computable visual features were extracted for each movie frame. The values were then averaged across 25-frame (i.e., 1 s) chunks. This was done because faster changes would probably not be reflected prominently in an adjustment of the rating slider.

#### DNN-based measures of visual fluency

This set of predictors features three measures: Part-similarity, integration, and L2 norm, all derived from DNN representations^33^. We employed a VGG16 deep convolutional neural network^41^ trained for scene classification on the Places365 dataset^42^. The pretrained network weights were obtained from the official Places365 repository and were originally implemented in Caffe^43^. The model was subsequently imported into MATLAB using the Deep Learning Toolbox Caffe importer.

We presented the network with either the full movie frame or spatially partitioned versions of the frame. Because the VGG16 network we used requires square image inputs, each movie frame was resized from its original resolution (1280 × 720 pixels) to 640 × 640 pixels. The partitions were then generated by dividing each resized frame into an 8 × 8 grid of equal-sized squares. Two complementary image sets were created: one containing all odd-numbered squares and the other containing all even-numbered squares, analogous to the black and white fields of a checkerboard pattern. The full image and both partitioned versions were fed separately into the network, and layer-specific activation patterns were extracted from the 11th layer, as previous work indicated that this layer yields the most robust predictions of aesthetic appeal^33^.

Part-similarity was quantified by correlating the activation patterns of the two complementary halves, describing how much different regions of an image resemble one another. Integration was assessed by averaging the activation vectors of the two halves and correlating this mean activation with the activation elicited by the full image, which defines how well these regions combine into a coherent whole. The resulting correlation values were sign-inverted so that weaker similarity between parts and whole reflected higher integration. Finally, L2 norms were computed by summing the squared activations of all units in the selected layer to quantify overall activation strength. Fig. S4 shows a conceptual illustration of the three DNN-based measures.

#### Symmetry

Symmetry was quantified by calculating pixel-wise correlations across all possible horizontal and vertical axes of reflection. For each image, the maximum correlation value, weighted by the number of pixels contributing to the computation, was taken as the measure of horizontal and vertical symmetry^36^.

#### Color statistics

Colorfulness, greenness, and blueness were computed in CIELab color space. For each frame, we extracted the *a* (green-red) and *b* (blue-yellow) channels. The mean and standard deviation of the *a* and *b* channels were computed across all pixels to quantify chromatic distribution. The colorfulness was defined as the Euclidean norm of the *a* and *b* standard deviations plus 0.3 times the Euclidean norm of their mean values, capturing both chromatic variability and overall color intensity^39^. Greenness and blueness were defined as sign-inverted versions of the averages of the *a* and *b* channels, respectively.

$${Colorfulness}=\sqrt{\left\{{\sigma }_{a}^{2}+\,{\sigma }_{b}^{2}\right\}}+0.3\sqrt{\left\{{\mu }_{a}^{2}+\,{\mu }_{b}^{2}\right\}}$$

Mean saturation was computed as the average of the HSL color space saturation values across all pixels for each frame. Hue contrast was computed following Iiagaya et al. (2021). Pixels with low saturation or extreme brightness were excluded, and the remaining pixels were assigned to bins on a circular hue histogram. Hue contrast was defined as the angular distance of the dominant hue bins on the hue circle. Because hue is a circular variable, if the largest angular gap between dominant hue bins exceeded π, the angular distance (2π − gap) was used. Larger values indicate a broader range of dominant hues in the image^40^. Brightness contrast was computed in RGB color space as the width of the smallest symmetric histogram interval centered on the most frequent brightness bin that contained 98% of all pixels^4^.

#### Motion energy

Motion energy was quantified using a spatiotemporal Gabor filter model, implemented with the publicly available MATLAB code from “https://github.com/gallantlab/motion_energy_matlab”^44^. The motion data was transformed using Principal Component Analysis (PCA) to capture its main patterns of variation. Only the first principal component is used, representing the dominant motion signal, which is then stored as a 1D motion energy predictor.

### Ridge regression models

To predict continuous aesthetic ratings from visual features, we applied ridge regression separately for each participant. We implemented a fully exhaustive nested cross-validation procedure across all movie segments (nine segments for *Home*, and eight segments for *Loving Vincent*). For each fold, one segment served as the test set, one segment as the validation set for hyperparameter selection, and the remaining segments as the training set. This resulted in 72 folds per participant (9 test segments × 8 validation segments) for *Home*, and 56 folds for *Loving Vincent* (8 test segments × 7 validation segments). Within each fold, predictor variables were standardized based on the training data. Ridge models were fitted on the training set for each λ value, and the optimal λ was chosen from a logarithmically spaced range (10⁻⁵ to 10⁵, 100 values) by minimizing mean squared error on the validation set. The final model with the selected λ was then evaluated on the held-out test set.

Model performance was quantified using Pearson correlation between predicted ratings and observed ratings for each segment. Performance was averaged across all folds per participant for both movies. At the group level, mean performance across participants was tested against zero using one-sided (right-tailed) one-sample *t* tests. For all *t* tests, effect sizes are reported as Cohen’s *d* with corresponding 95% confidence intervals. To examine predictor contributions, regression coefficients were averaged across folds within each participant. Group-level significance of coefficients was assessed using one-sample *t* tests across participants, with false discovery rate (FDR) correction applied to account for multiple comparisons. Assumptions of the parametric statistical analyses were assessed using Anderson–Darling tests and visual inspection of Q–Q plots. The participant-level data used in the one-sample *t* tests showed no substantial deviations from normality.

### Cross-participant predictions

To assess cross-subject generalization, we trained ridge regression models to predict leave-one-subject-out group-mean rating time courses from the selected predictors and then evaluated these models against the ratings of the left-out participant. For each left-out participant and movie segment, the training target was the mean rating time course of the remaining *N*-1 participants. The regularization parameter (*λ*) was selected separately for each participant and model using leave-one-segment-out cross-validation: in each fold, the model was trained on all but one segment and validated on the held-out segment by minimizing mean squared error. Using the optimal *λ*, the final model was fit across all segments and then used to predict the left-out participant’s moment-to-moment ratings for each segment. Prediction accuracy was quantified as the Pearson correlation between predicted and observed ratings, and correlations were averaged across segments to obtain a single performance estimate per participant. Group-level significance was assessed using one-sided (right-tailed) one-sample *t* tests across participants, with false discovery rate (FDR) correction.

### Assessing the relative contribution of features

The same ridge regression procedure was repeated with reduced models, excluding one predictor group each time. Then, the correlations of predicted and observed ratings for each reduced model were subtracted from the correlations obtained with the full model. We conducted a one-tailed *t* test with FDR correction to compare whether the predictor group significantly reduced the model performance and contributed to predicting aesthetic preferences for both movies.

### Cross-movie predictions

Next, we examined whether models trained on aesthetic ratings for one type of content could predict ratings for a different type of content. For this purpose, we created two ridge regression models. In each case, models were trained on *N*-1 segments of the average ratings and predictors from one movie (e.g., *Home*), using the remaining segment for regularization, and then retrained on all *N* segments. The final model was subsequently tested on each segment of each participant from the other movie (e.g., *Loving Vincent*). The same procedure was applied in the reverse direction (training on *Loving Vincent* and testing on *Home*). Further, we again performed model comparison analyses to examine how cross-dataset predictions are driven by the different predictor groups.

## Results

In Experiment 1, a total of 37 participants viewed the naturalistic movie *Home*, which was divided into nine segments (mean duration = 505.1 seconds). In Experiment 2, 30 participants viewed the artistic movie *Loving Vincent*, which was divided into eight segments (mean duration = 581.3 s).

For both movies, we extracted visual feature descriptors from each frame and resampled them to a one-second resolution to align with moment-to-moment aesthetic appeal ratings. These visual features were used as predictors to model aesthetic appeal ratings. A total of twelve predictors were extracted, split into four groups: visual fluency, symmetry, color, and motion (Fig. 2; see “Methods” for details).

### Visual features robustly predict aesthetic appeal ratings

**Fig. 3:**
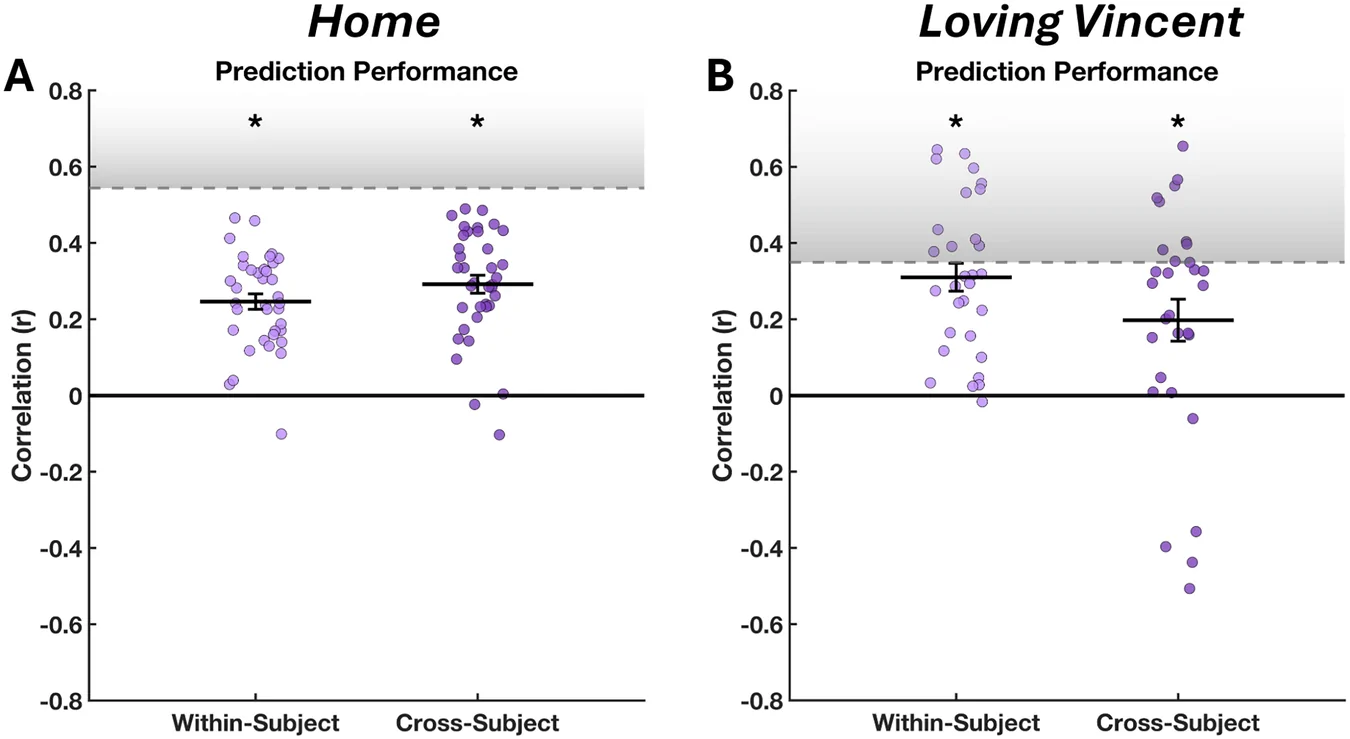
Within- and cross-subject prediction performance. For both (**A**) *Home* (Experiment 1, *N* = 37) and (**B**) *Loving Vincent* (Experiment 2, *N* = 30), the grey area indicates the noise ceiling, representing the maximum achievable prediction performance, computed by correlating each subject’s ratings with the average ratings of all other subjects across movie segments. Dots represent predictive performance in individual participants, quantified as the correlation between predicted and observed ratings. Error bars indicate SEM. *P* values were obtained from one-sided (right-tailed) one-sample *t* tests against zero; asterisks indicate *p* < 0.05 after FDR correction for multiple comparisons.

For each participant, we then used a cross-validation approach across movie segments to predict moment-to-moment aesthetic ratings from visual features. In all analyses, linear models were trained to predict aesthetic appeal ratings from the visual predictors on a subset of the movie segments and evaluated on a left-out movie segment. Specifically, for each cross-validation fold, one segment was held out as the test set, a second segment served as a validation set, and the remaining segments (seven for *Home*; six for *Loving Vincent*) were used for training. A ridge regression model was trained to predict aesthetic ratings (Y) from the four predictor groups (twelve visual predictors) (X), including visual fluency, symmetry, color statistics, and motion energy. Models were fitted on the training set with a range of regularization parameters λ, and predictive performance was evaluated on the validation set for each. We then selected the λ with the best performance on the validation set. Finally, a model with this optimal λ was fitted on the training set and in turn evaluated on the held-out test set. Model performance was quantified by computing the Pearson correlation between predicted and observed aesthetic appeal ratings. The model significantly predicted aesthetic appeal ratings in both *Home* (*t*(36) = 12.18, *p* < 0.001, Cohen’s *d* = 2.03, *CI* = [1.46, 2.59]) and *Loving Vincent* (*t(*29) = 8.48, *p* < 0.001, Cohen’s *d* = 1.58, *CI* = [1.03, 2.11]) (Fig. 3). These findings indicate that visual features predict dynamic aesthetic experiences and that predictions are robust for different visual content.

### Visual features predict aesthetic appeal across participants

The previous analysis, in which models are trained and tested within individual participants, demonstrates that visual features predict aesthetic appeal ratings within individuals. Next, we asked whether these relationships generalize across individuals, which would indicate that participants evaluate visual features in similar ways. To assess cross-subject generalization, we trained ridge regression models to predict group-level aesthetic dynamics from visual features and evaluated them on individual left-out participants. The regularization parameter (λ) was selected using leave-one-segment-out cross-validation on the group-mean data. In this cross-participant analysis, the model still showed significant predictive performance for both *Home* (*t(*36) = 12.35, *p* < 0.001, Cohen’s *d* = 2.06, *CI* = [1.48, 2.63]) and *Loving Vincent* (*t(*29) = 3.58, *p* = 0.001, Cohen’s *d* = 0.66, *CI* = [0.26, 1.06]) (Fig. 3). These findings demonstrate that visual feature models yield predictions that generalize across participants, suggesting that visual features contribute to aesthetic appeal in similar ways for different individuals. Yet cross-participant predictions were more accurate for *Home* than for *Loving Vincent*, consistent with greater individual variability in the aesthetic appeal of artistic stimuli^45^.

### Visual fluency and color statistics drive predictions of aesthetic experiences

After establishing that visual features predict aesthetic experience within and across participants, we next asked which features contribute most to this prediction. To address this, we conducted a model comparison analysis in which models were retrained while leaving out one predictor group (i.e., fluency, symmetry, color, motion) at a time. The change in performance (Δr) was computed by subtracting the reduced models’ predictive performance from the full model’s performance. We then performed one-sample right-tailed *t* tests to determine whether excluding each predictor group significantly reduced model performance.

**Fig. 4:**
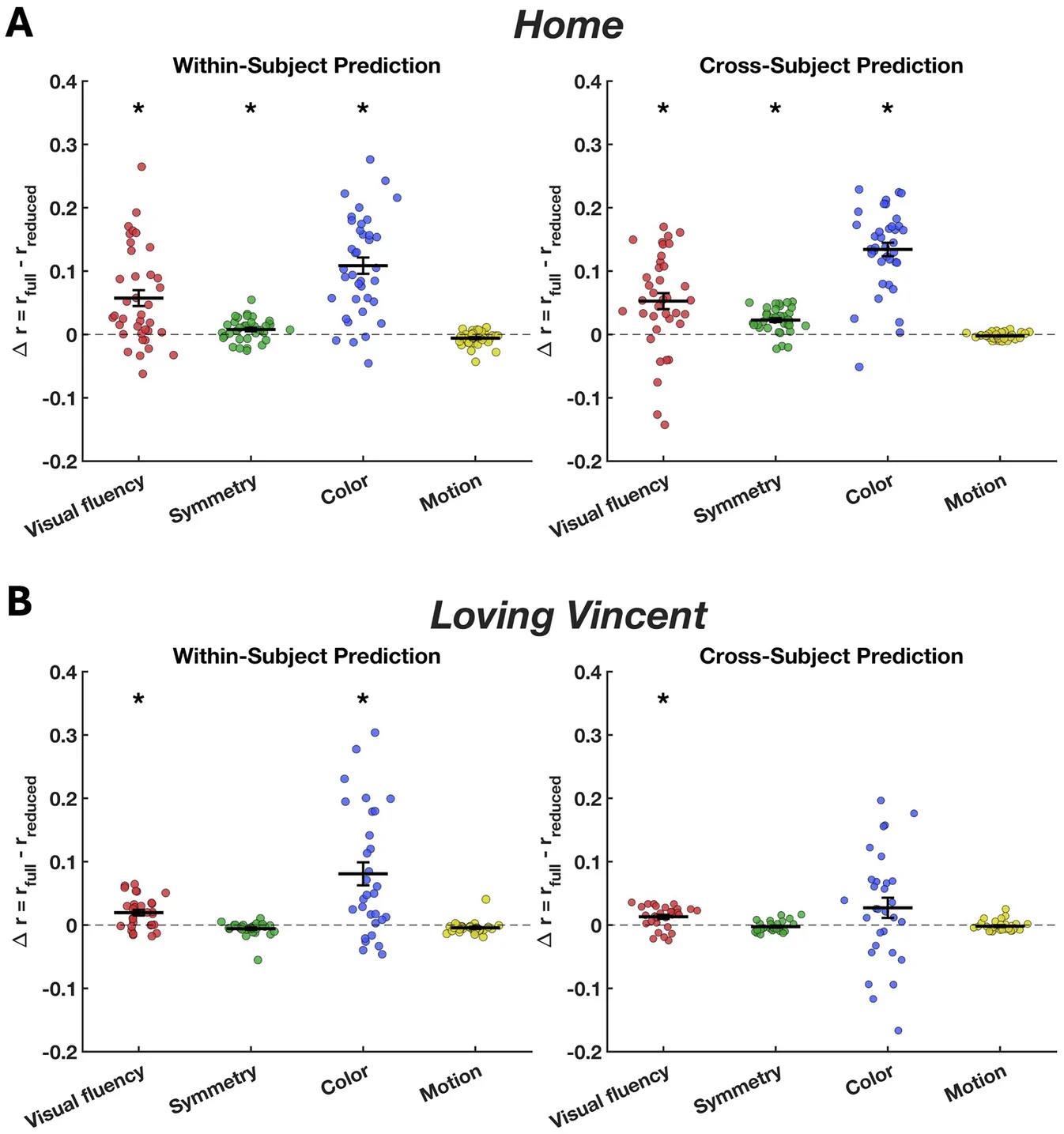
Model comparison results. The contribution of each predictor group to the performance of the full model is shown for both within- and cross-subject analyses. The y-axis represents the change in predictive performance (Δr), computed as the difference between the full and reduced models. (**A**) For *Home* (Experiment 1, *N* = 37), visual fluency, symmetry, and color predictors significantly reduced model performance in both within- and cross-participant analyses. (**B**) For *Loving Vincent* (Experiment 2, *N* = 30), leaving out visual fluency and color predictors reduced model performance in both within- and cross-participant analyses. Error bars indicate SEM. *P* values were obtained from one-sided (right-tailed) one-sample *t* tests against zero; asterisks indicate *p* < 0.05 after FDR correction for multiple comparisons.

For *Home*, both within- and cross-participant analyses showed that leaving out visual fluency (within: *t*(36) = 4.59, *p* < 0.001, Cohen’s *d* = 0.77, *CI* = [0.39, 1.13]; cross: *t*(36) = 4.17, *p* < 0.001, Cohen’s *d* = 0.69, *CI* = [0.33, 1.05]), symmetry (within: *t*(36) = 2.76, *p* = 0.006, Cohen’s *d* = 0.46, *CI* = [0.11, 0.80]; cross: *t*(36) = 7.24, *p* < 0.001, Cohen’s *d* = 1.21, *CI* = [0.77, 1.63]), and color (within: *t*(36) = 8.43, *p* < 0.001, Cohen’s *d* = 1.41, *CI* = [0.94, 1.86]; cross: *t*(36) = 12.62, *p* < 0.001, Cohen’s *d* = 2.10, *CI* = [1.51, 2.68]) significantly reduced model performance, while leaving out motion (within: *t*(36) = –3.25, *p* = 0.1, Cohen’s *d* = –1.8, *CI* = [–1.78, –0.38]; cross: *t*(36) = –2.76, *p* = 0.1, Cohen’s *d* = –0.92, *CI* = [–1.60, –0.23]) did not significantly reduce model performance (Fig. 4A). For *Loving Vincent*, leaving out visual fluency (within: *t*(29) = 4.34, *p* < 0.001, Cohen’s *d* = 0.81, *CI* = [0.38, 1.22]; cross: *t*(29) = 4.15, *p* < 0.001, Cohen’s *d* = 0.77, *CI* = [0.35, 1.18]) and color (within: *t*(29) = 4.44, *p* < 0.001, Cohen’s *d* = 0.83, *CI* = [0.40, 1.24]; but cross: *t*(29) = 1.7, trending at *p* = 0.10, Cohen’s *d* = 0.32, *CI* = [–0.06, 0.69]) reduced model performance, while leaving out symmetry (within: *t*(29) = –2.86, *p* = 0.10, Cohen’s *d* = –1.06, *CI* = [–1.83, –0.28]; cross: *t*(29) = –1.74, *p* = 0.09, Cohen’s *d* = –0.65, *CI* = [–1.39, 0.11]) and motion (within: *t*(29) = –2.46, *p* = 0.10, Cohen’s *d* = –0.91, *CI* = [–1.67, –0.14]; cross: *t*(29) = –1.17, *p* = 0.09, Cohen’s *d* = –0.43, *CI* = [–1.17, 0.31]) did not significantly reduce model performance (Fig. 4B).

### Visual features predict aesthetic appeal across different movie content

Next, we tested whether models trained on one movie can predict aesthetic appeal ratings in the other movie, despite the marked differences in content and style across the two movies. This analysis provides a critical test of how visual features affect aesthetic appeal across domains. Here, models were trained on *N*-1 segments of the average ratings and predictors from one movie (e.g., *Home*), using the remaining segment for regularization, and then retrained on all *N* segments. The final model was subsequently tested on each segment of each participant from the other movie (e.g., *Loving Vincent*).

**Fig. 5:**
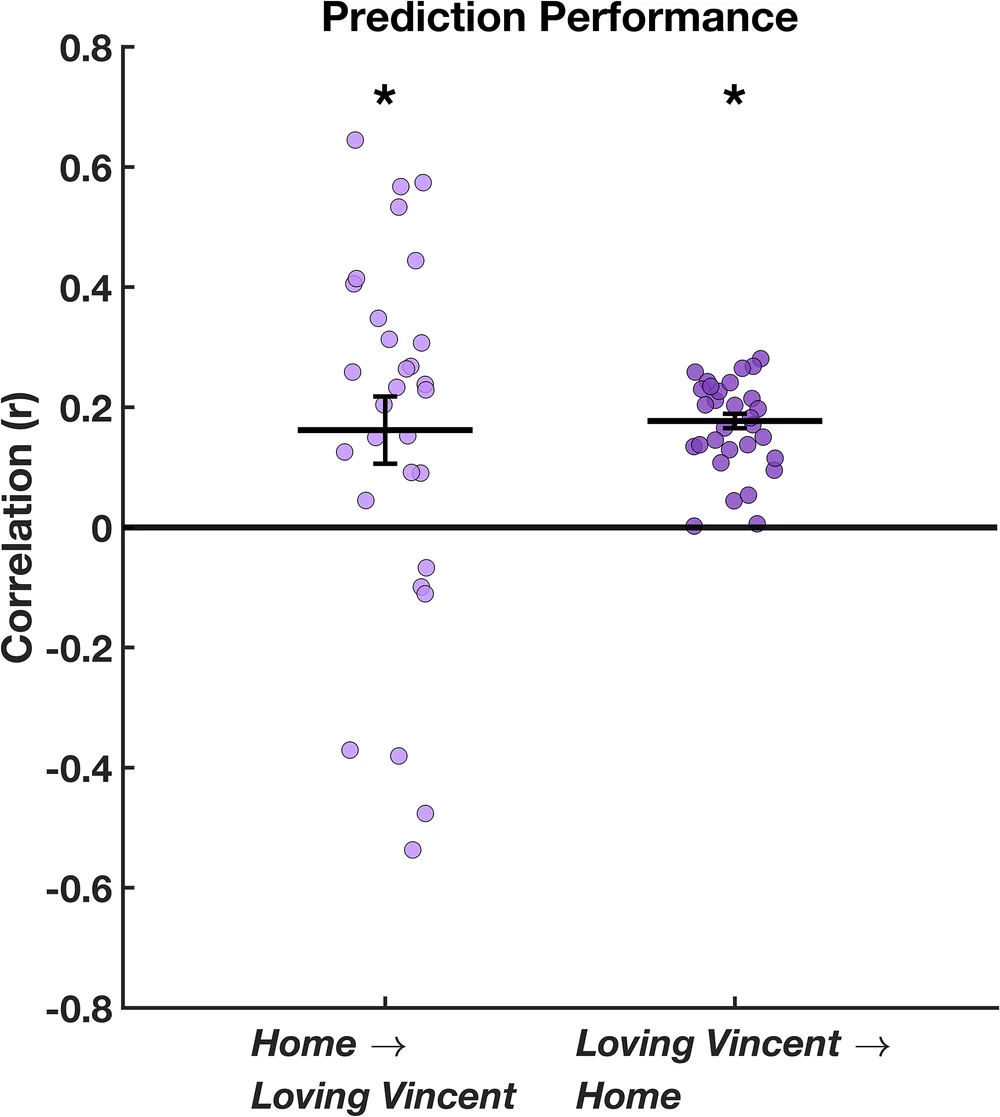
Cross-dataset prediction performance. Models were trained on the average aesthetic ratings of *Home* (Experiment 1, *N* = 37) and tested on individual participants in *Loving Vincent* (Experiment 2, *N* = 30), and vice versa. Predictive performance is shown as the correlation between predicted and observed ratings. Error bars indicate SEM. *P* values were obtained from one-sided (right-tailed) one-sample *t* tests against zero; asterisks indicate *p* < 0.05 after FDR correction for multiple comparisons.

Models trained on one movie significantly predicted aesthetic appeal ratings in the other movie, in both train-test directions (*Home* to *Loving Vincent*: *t*(29) = 2.89, *p* = 0.007, Cohen’s *d* = 0.54, *CI* = [0.15, 0.92]; *Loving Vincent* to *Home*: *t*(36) = 14.76, *p* < 0.001, Cohen’s *d* = 2,46, *CI* = [1.80, 3.11]) (Fig. 5). Predictive performance was less variable across individuals when the model was trained on *Loving Vincent*. This asymmetry may reflect differences in the structure of the visual feature space across the two movies. Specifically, *Loving Vincent* occupies a lower-dimensional feature space than *Home*, meaning that its visual content can be described by fewer independent combinations of visual features. Indeed, a principal component analysis of the extracted visual predictors supported this interpretation: the first principal component explained a larger proportion of the variance in *Loving Vincent* (40.8%) than in *Home* (23.9%), and only six principal components were required to explain 90% of the variance, compared with eight for *Home* (Figure S5).

### Cross-movie predictions are predominantly driven by color features

To further examine the contribution of each predictor group to this cross-dataset generalization, we again performed a model comparison analysis. For each train-test direction, models were evaluated while leaving out one predictor group at a time. Prediction performance was again quantified as the correlation between predicted and observed ratings, and the change in performance (Δr) was computed by subtracting the reduced model performance from the full model performance. One-sample right-tailed *t* tests were used to assess whether excluding each predictor group significantly reduced prediction performance.

**Fig. 6:**
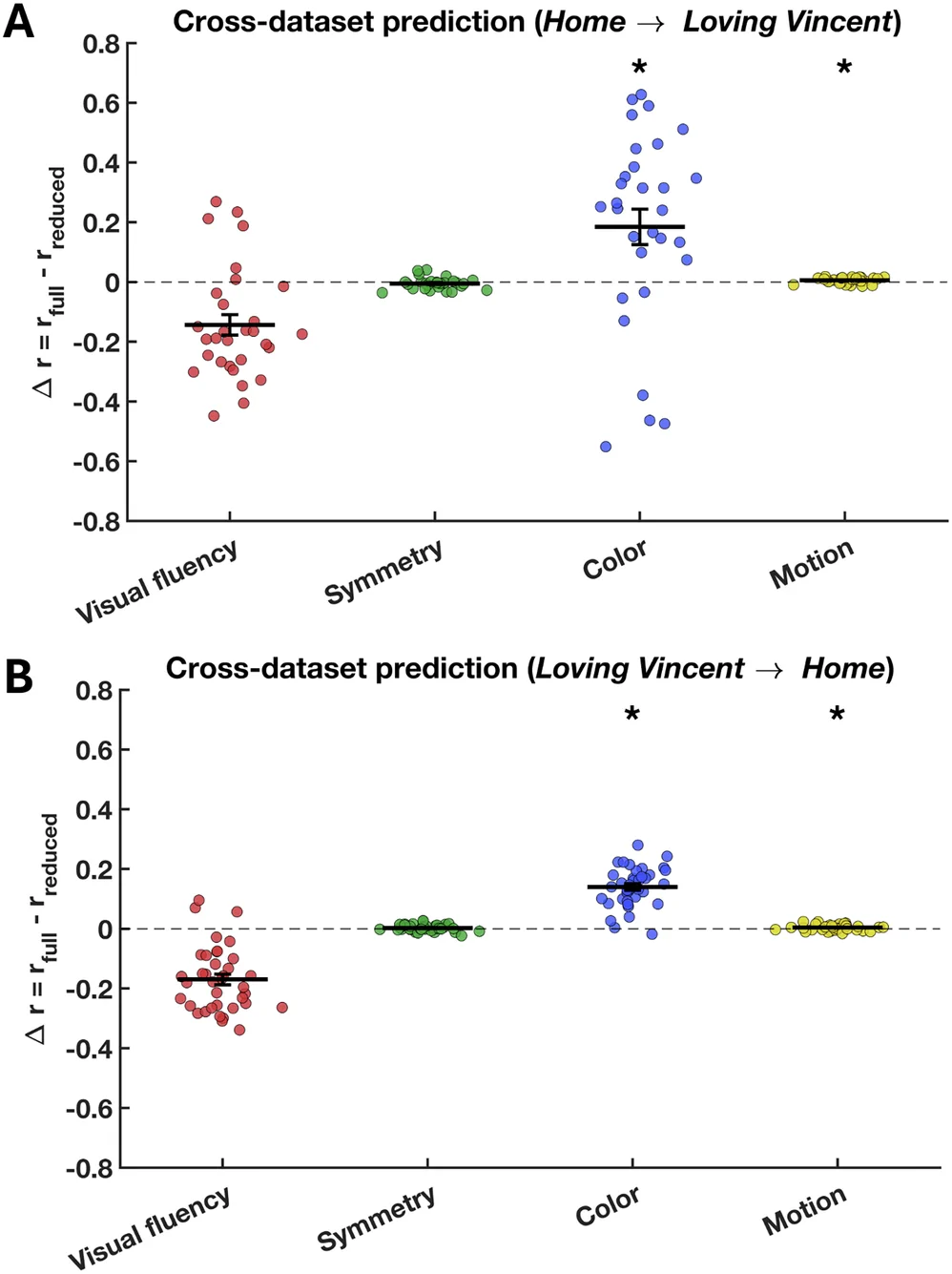
Cross-dataset prediction performance using model comparison. The contribution of each predictor group to the performance of the full model is presented for cross-dataset prediction. The y-axis shows the change in prediction performance (Δr), computed as the difference between the full and reduced model performances. (**A**) When training the models on *Home* (Experiment 1, *N* = 37) and testing them on *Loving Vincent* (Experiment 2, *N* = 30), only color statistics significantly reduced model performance, whereas other predictor groups did not. (**B)** When training the models on *Loving Vincent* and testing them on *Home*, color statistics and motion energy significantly reduced model performance, whereas visual fluency and symmetry did not. Error bars indicate SEM. *P* values were obtained from one-sided (right-tailed) one-sample *t* tests against zero; asterisks indicate *p* < 0.05 after FDR correction for multiple comparisons.

When models were trained on the average aesthetic appeal ratings from *Home* and tested on the individual ratings in *Loving Vincent*, model comparison analysis showed that leaving out color (*t*(29) = 3.12, *p* = 0.004, Cohen’s *d* = 0.58, *CI* = [0.18, 0.97]) and motion (*t*(29) = 3.08, *p* = 0.004, Cohen’s *d* = 0.57, *CI* = [0.18, 0.96]) predictors significantly reduced model performance, whereas visual fluency (*t*(29) = –4.22, *p* = 0.10, Cohen’s *d* = –1.57, *CI* = [–2.39, –0.73]) and symmetry (*t*(29) = –1.56, *p* = 0.10, Cohen’s *d* = –0.58, *CI* = [–1.32, 0.17]) did not significantly reduce model performance (Fig. 6A). When the models were trained on the average ratings from *Loving Vincent* and tested on the individual ratings from *Home*, leaving out both color (*t*(36) = 12.8, *p* < 0.001, Cohen’s *d* = 2.13, *CI* = [1.54, 2.72]) and motion (*t*(36) = 2.53, *p* = .02, Cohen’s *d* = 0.42, *CI* = [0.08, 0.76]) predictors reduced model performance, whereas visual fluency (*t*(36) = -9.53, *p* = 1, Cohen’s *d* = –3.18, *CI* = [–4.15, –2.19]) and symmetry (*t*(36) = –1.44, *p* = 0.10, Cohen’s *d* = –0.48, *CI* = [–1.14, 0.19]) did not (Fig. 6B).

Together, these results indicate that color statistics provide the most consistent contribution to cross-dataset prediction, suggesting that they capture shared constraints on aesthetic experience across visually and stylistically distinct movies. Excluding the motion predictor also yielded a significant reduction in performance. However, the effect was numerically very small, and motion did not consistently contribute to predictions within individual movies. The effects observed here may thus stem from systematic but weak interactions with other predictors. Interestingly, while the visual fluency predictor group was informative for predictions within each movie, it did not generalize to the cross-dataset analysis. Specifically, in the naturalistic movie *Home*, similarity and integration yielded positive regression weights, whereas L2 norm yielded a negative weight, consistent with previous findings on natural scenes^33^. In contrast, in the artistic movie *Loving Vincent*, L2 norm yielded positive weights, while similarity and integration yielded negative weights (Fig. S3). This may indicate differences in how visual fluency contributes to aesthetic experience across different types of visual content.

## Discussion

Our study demonstrates that aesthetic experience can be robustly predicted from visual features. By combining multiple low- and mid-level image-computable features, we modeled moment-to-moment fluctuations in perceived beauty across dynamic movie stimuli. These visual-feature models not only predicted aesthetic appeal ratings on an individual level; trained models also predicted aesthetic appeal across different observers and across the two movies. Our findings thus support the idea that aesthetic experience is rooted in perceptual processes, which play out in similar ways across individuals and different content.

### Visual features predict aesthetic appeal across observers

The finding that model predictions generalize across observers suggests that aesthetic judgments are not purely idiosyncratic but partly grounded in shared visual feature preferences. This finding challenges the common assumption that “beauty lies in the eye of the beholder,” instead pointing to a form of “perceptual” beauty that is shared across individuals. At the same time, predictions across observers varied with content: cross-participant predictive performance was reduced for *Loving Vincent* compared to *Home*, which suggests that the aesthetic appeal of artistic stimuli is more variable across individuals. This finding aligns with prior work showing that artworks and architecture tend to evoke more disagreement among people than natural scenes, likely due to differences in interpretation, exposure, and cultural artifacts^45^. Yet, reduced cross-participant predictions may not only relate to higher-level cognitive differences but also to individual variability in how visual features are mapped onto aesthetic appeal: Studies on the neural representation of aesthetic appeal indicate that even early brain responses associated with perceptual processing are partly idiosyncratic^46,47^.

### Color predicts aesthetic appeal across different content

Color statistics and visual fluency were the most informative predictors both within and across participants, whereas symmetry and visual motion played a comparatively minor role. Particularly, color features were the only predictors that robustly generalized across movies. Among the color predictors we used, overall variation in colors (colorfulness), along with brightness contrast, contributed most to successful predictions of aesthetic ratings (Fig. S3), consistent with prior work showing that colorfulness is a significant predictor of aesthetic preference^48^. The positive contribution of colorfulness (positive beta coefficient) indicates a preference for colorful visual inputs. One possible explanation is that color conveys not only low-level visual information but also higher-level ecological and emotional cues. According to the Ecological Valence Theory (EVT) of color preference, preferences for specific colors arise because they are associated with positively or negatively valued objects and environments rather than because of the colors themselves^37^. Moreover, our results indicate that green and blue hues are associated with greater aesthetic appeal than red and yellow hues (Fig. S3). Consistent with this pattern, earlier work has shown that bluish and greenish color ranges tend to be preferred over darker or brownish tones in aesthetic evaluations of paintings^49^. According to the EVT, the positive contributions of green and blue hues in our models may reflect the positive affective associations that these colors acquire through experience with natural environments^37^. Specifically, blue and green are commonly associated with aesthetically preferred natural elements such as skies, water, and rich forests. Thus, rather than reflecting an isolated preference for these hues alone, their contribution to aesthetic appeal may partly arise from the positive ecological and semantic information they convey. Color may therefore play an efficient role that more broadly captures perceptual properties of a scene and aspects of its semantic and emotional content, potentially explaining why color-related features generalized more robustly across two visually and semantically distinct movies than the other visual features considered here. This is in line with a previous study showing that preferences for color compositions persist even when spatial structure and semantic content are disrupted, such as in scrambled or patchwork images^50^. Interestingly, the same study reported only weak and non-replicable cultural differences, suggesting that the dominance of color statistics in aesthetic preference is robust across observers. This notion is consistent with our cross-movie predictions being primarily driven by color features and indicates that color can serve as a reliable predictor of aesthetic appeal despite substantially higher-level differences in context, content, and cultural interpretation. The importance of color for aesthetic appeal is potentially rooted in biological or ecological factors, such as sensitivity to natural color distributions^51^. Notably, our finding that color was an important predictor across two movies should not be interpreted as implying that color is necessary for aesthetic experience. Rather, our findings suggest that, when color information is present, it provides particularly informative cues for predicting dynamic aesthetic preferences. Supporting this interpretation, an exploratory analysis of *Loving Vincent*, which contains both colorful and (largely) monochromatic scenes (40.8% of the movie), showed that scenes with minimal color information received significantly lower aesthetic ratings than colorful scenes (*t*(29) = − 2.28, *p* = 0.03, Cohen’s *d* = –0.42, *CI* = [–0.8, –0.04]). Thus, when chromatic and achromatic scenes coexist within the same movie, chromatic scenes elicit significantly higher aesthetic ratings, further supporting the contribution of color to aesthetic experience. However, in the absence of color, as in Charlie Chaplin films or Magnum Photos, other visual features, such as luminance, texture, and motion, as well as higher-level factors including narrative meaning and emotional engagement, may play a greater role in shaping aesthetic appeal.

### Fluency predicts aesthetic appeal in nuanced ways

Although visual fluency was predictive within each movie, it showed qualitatively different contributions across the two movies. In the naturalistic movie *Home*, fluency-related measures aligned with previous findings on natural scenes: similarity and integration contributed positively to the predictions, indicating that efficient integration of image parts predisposes greater aesthetic appeal, whereas the L2 norm showed a negative contribution, indicating that representational sparseness predisposes greater aesthetic appeal^33,52^. However, in *Loving Vincent*, the contribution of these three predictors was reversed (Fig. S3), potentially indicating that greater fluency yields *lower* aesthetic appeal. This divergence indicates that fluency effects may be modulated by the statistical structure and style of the visual input. One possible explanation is that more artistic depiction (like the painting style in *Loving Vincent*) engages a different mode of aesthetic evaluation, similar to the processing of artworks. In this mode, observers may shift from rapid, perceptually driven judgments to more reflective processing that enables them to understand or interpret more deeply and become more sensitive to complexity, ambiguity, and higher-level features^53^. In this reflective mode, less fluent inputs may be more attractive, as they provide a richer basis for engagement and interpretation. Another explanation is that the deep neural network used to derive these features (VGG16 trained on Places365) is trained on natural images rather than artworks^54^ and may therefore capture fluency in a way that is better aligned with natural scene statistics than with painterly representations. Regardless, it is important to note that fluency was a powerful predictor for aesthetic appeal in both movies. Yet, its role may depend on the context or the representational format of the stimulus.

### How “good” are models based on interpretable visual features?

Despite the success of our feature-based prediction models, a considerable portion of variance in aesthetic ratings remains unexplained. This likely reflects the influence of higher-level factors, such as semantic meaning, emotional engagement, cultural differences, and individual differences in personality or prior experience^55^. In particular, our models focused on image-computable visual features of low to intermediate complexity and therefore did not explicitly capture differences in semantic content or narrative structure, which likely also contribute to dynamic aesthetic experiences. At the same time, our approach can be considered a starting point, where the inclusion of other, more complex feature predictors could further push the boundaries of predictive accuracy. In this context, it is important to note that our analysis deliberately focused on a set of relatively simple and interpretable predictors. It is possible that more comprehensive feature spaces could yield improved predictions^56^. Indeed, recent work has shown that “brute-force” approaches, directly regressing deep neural network (DNN) activity patterns onto aesthetic ratings^33,57^, achieve high predictive performance. However, the models employed in such studies are essentially black boxes, with regressions on high-dimensional spaces of arbitrary DNN features offering limited insight into the specific features driving aesthetic experiences. Our approach is complementary to that, emphasizing interpretability to identify which visual properties meaningfully contribute to perceived beauty. Finally, it is important to emphasize that these findings are based on two visually and semantically distinct movies. Although successful predictions across these two distinct movies support the validity of our findings to some extent, a broader range of visual styles and semantic content is necessary to establish the generality of the identified feature–aesthetic relationships and determine whether color statistics play a dominant role in dynamic aesthetic experiences more broadly.

### Looking forward: continuous ratings of aesthetic appeal as an emerging method

The present study highlights the value of using dynamic, naturalistic stimuli to investigate aesthetic experiences, while allowing participants to provide continuous, moment-to-moment ratings of aesthetic appeal. Importantly, our findings demonstrate that principles established in studies using static images and individual ratings generalize to complex, dynamic stimuli assessed with continuous measures. This suggests that core perceptual drivers of aesthetic experience remain stable across stimulus formats and timescales. At the same time, our findings further showcase the viability of studying aesthetic experiences with continuous ratings, offering an ecologically valid and temporally fine-grained account of aesthetic experience^58^. The continuous rating approach provides opportunities for investigating the temporal dynamics of aesthetic experience^59^. For instance, it can be used to link fluctuations in aesthetic appeal to fluctuations in neural dynamics during movie watching or to test predictive models of how aesthetic appeal unfolds over time^60,61^.

### Limitations

Two limitations of our study warrant further attention. First, our findings are based on only two movies. Although these movies differ a lot in both content and visual style, they cannot capture the full diversity of naturalistic dynamic visual experiences. Our findings should be interpreted as evidence that visual-feature models generalize across two very distinct movies and across viewers, rather than as demonstrating universal feature preferences. Future work needs to examine a wider range of movies, genres, and visual contents to determine the extent to which these feature preferences generalize across naturalistic contexts. Second, the continuous rating paradigm used here may have emphasized perceptual processing, with movie watching and rating reports putting additional load on cognitive resources. This may tip the balance between rapid, automatic perceptual processing and slower, controlled cognitive processing involved in aesthetic appreciation^8,62^. While we cannot exclude that this tipped balance leads to an overestimation of perceptual factors, the differences may not be so pronounced in practice: As Isik & Vessel (2019) reported previously, preference judgments for short videos do not differ systematically between continuous “online” ratings and “offline” ratings after the movie presentation.

## Conclusion

In sum, our findings suggest that aesthetic experience during movie watching is partly related to shared perceptual mechanisms captured by visual features, while individual differences and contextual factors continue to play an important role. Across two visually distinct movies, these feature-based relationships generalized across observers and movie content, highlighting the contribution of perceptual processing to dynamic aesthetic experience. Future research should include a broader range of visual content and integrate low-level feature-based approaches with higher-level cognitive and affective factors to develop a more comprehensive model of aesthetic experience.

## Supplementary information

Transparent Peer Review file

Supporting Information

## Data Availability

Data are available through the project’s OSF page (https://osf.io/vcuj7)^63^.

## Code availability

Code is available through the project’s OSF page (https://osf.io/vcuj7)^63^
