# Supplementary material for: Visual features explain dynamic aesthetic experiences across distinct movie content
Source: Commun Psychol. 2026 Sep 11;4:127. doi: 10.1038/s44271-026-00531-7 (PMC13569840; doi:10.1038/s44271-026-00531-7)
Supplement: Supplementary file 1 [file 44271_2026_531_MOESM1_ESM.pdf]

nature portfolio

Peer Review File

Visual features explain dynamic aesthetic experiences across distinct movie content

Corresponding Author: Mr Mustafa Alperen Ekinci

This file contains all reviewer reports in order by version, followed by all author rebuttals in order by version.

Version 0:

Reviewer comments:

Reviewer #1

(Remarks to the Author)
The authors of this manuscript endeavor to identify the visual features underlying the aesthetic evaluation of movies. The presented participants with two quite different movies while participants were asked to continuously rate their aesthetic enjoyment. The authors then analyze the feature content of the movies in feature groups of “visual fluency”, “symmetry”, “color statistics”, and “visual motion”. Using Ridge Regression, they then predict participants’ aesthetics ratings both within and across subjects, and both within and across movies. They find color statistics to be the most influential feature for predicting aesthetic liking.

The study is well designed, the analysis is well motivated, and the paper is well written. I have a few comments and questions..

1. My main issue with the manuscript is that this seems fairly thin data on which to stake bold claims. The two movies shown here are quite different, but each is also very specific in content and visual features. How can we know that the main claim of the paper – that color statistics explain dynamic aesthetic experiences – holds in general? Yes, the main finding replicates across data sets. In the end, I believe that the authors can really only make claims about those two particular movies. The authors are cautious not to overstate their results. Yet, the stimulus-specificity of the results limits the broader impact of the findings.

2. The authors correctly emphasize the differences in visual features between the two movies (e.g., in the 5th paragraph of the introduction, at the bottom of page 2). I strongly recommend that they should also comment on the differences in content, which will likely affect aesthetics ratings to a large degree.

3. Page 9, second paragraph: The authors observe an asymmetry in the variability of cross-movie predictions. Their explanation (sentence starting with “This may reflect …”) is a bit nebulous. I would encourage the authors to explore this issue further, ideally with additional analyses of the properties of the data sets. I believe that the asymmetry is a significant finding that deserves a bit more attention and interpretation.

4. Page 12, 3rd paragraph (discussion of color): Why did black-and-white movies ever capture the audience’s attention? Why should green and blue be particularly important for aesthetic appeal? Do these hues correlate with particular content, such as landscapes, which are known to be aesthetically preferred across genres? I am looking for insights into aesthetics here. The way the discussion is written, the authors could have commented equally well on any other of the features to turn out to be important. What is it about color? Is it its relationship to emotions? Here is where the important scientific work needs to happen.

5. By comparison, fluency did not predict aesthetic preference well across movies (page 13, second paragraph). The authors go to great lengths to explain why this might be the case by invoking differences in movie content and emotionality. Why would these differences matter for fluency but not for other features? Emotions are strongly tied to colors, so why do these difference not appear in the color analysis?

6. I believe that point 5 above illustrates the issue that I raised in point 1. If the interpretation of the results requires detailed discussion of the idiosyncrasies of each of the movies, then perhaps two movies aren’t enough to make general statements.

It’s still a fine paper when the story is about these particular two movies, but its impact is not the same as if the authors could make more general claims.

7. One minor methods detail: Page 16, 5th paragraph: The movies are divided into 8x8 squares. Does that mean that the movies are cropped to a square for the experiment? If so, then this should be mentioned and justified.

Reviewer #2

(Remarks to the Author)
This study investigated how aesthetic appeal is perceived from videos. Participants continuously reported aesthetic appeal while watching movie clips, and their responses were compared with computational metrics describing video characteristics. The study made a progressive attempt to investigate aesthetic appeal along time series instead of static displays. It is interesting to see that the predictions generalized between two types of movies and across participants.
The fact that this study investigates time series leads to the first concern. The current analysis of human responses is at risk of losing substantial information and becoming misleading. To start with, the granularity of the recorded data is unclear. Participants continuously responded using a sliding bar. Although the responses were described as “moment-to-moment”, I highly doubt that analog signals were recorded. What was the sampling rate of human responses? A similar issue applies to the stimuli. The authors only indirectly mentioned that the videos had a frame rate of 25 fps and did not specify whether the videos were displayed at the same frame rate via Psychtoolbox.
The hazard lies in the following procedure. Although not overtly specified, I presume that human responses were binned by one-second slots in congruence with computational metrics, “because faster changes would probably not be reflected prominently in an adjustment of the rating slider.” This claim might be true, but it needs to be supported by empirical evidence instead of speculation. A sampling rate of 1Hz leads to a Nyquist frequency of 0.5Hz, which is one oscillation every 2 seconds. I can easily imagine participants moving the sliding bar at a higher frequency, and a sampling rate as low as 1Hz is likely to induce aliasing, which is an artifact that causes misrepresentation. To better represent human responses, the authors need to determine a range of relevant frequencies based on the raw data of human responses (which were presumably sampled at an excessively high rate), and bin their data by a time slot corresponding to twice the highest relevant frequency (see Nyquist–Shannon sampling theorem). The resulting time slot might end up being close to one second, but I would like to see evidence of this choice.
The second concern is also related to time series analysis. I believe that the authors binned computational metrics and human responses by one-second slots and treated each slot as an independent data point in the regression. This analysis diminishes temporal dependency, which is a crucial difference between videos and static displays. Also, the computational metrics were computed independently for each frame, i.e., information in stimuli was integrated spatially but not temporally. I speculate that the predictability of a given frame from previous frames would correlate with aesthetic appeal.
The third concern is that the authors made too strong a claim on the contribution of perceptual features to aesthetic appeal (“aesthetic experience is best understood as shared subjectivity”). The correlations between model predictions and observations are not strong (smaller than 0.4 in Figure 3 and smaller than 0.2 in Figure 5). Moreover, the current paradigm enhances the effects of perceptual factors. Compared with a common design that requires participants to respond after exposure to stimuli, the current design requires participants to complete two tasks simultaneously: watching the video and responding. The latter task divides attention. The PIA model (Graf & Landwehr, 2015) separates aesthetic processing into automatic and controlled processes and specifies that attention is required to enter the controlled process. Dividing attention inhibits the contribution of the controlled process, which involves “cognitive enrichment”. The same prediction can be made from another theoretical framework. Redies (2015) distinguished between the aesthetics of perception and the aesthetics of cognition. Simultaneous reports raise cognitive load, which inhibits the aesthetics of cognition and increases the contribution of the aesthetics of perception.
In addition to the concerns mentioned above, the methods are grossly underspecified. Please see the list of minor concerns below.
Minor concerns:
(1) In Figure 1, I am not sure what the tiny rating bars in the line plots stand for, and they do not seem to correspond with the line plot values. Please remove or specify.
(2) Please include a gross introduction on computational metrics in the introduction section, i.e., mentioning the contents in Figure 2. It is not clear what the “multiple visual properties” are and why they are important/relevant.
(3) In “Movie Stimulus”, why were there 8 randomized sequences in Experiment 1 and 18 in Experiment 2? The number of segments between the two movies was roughly the same. Please explain.
(4) In “Procedure”, did the participants respond by dragging a bar with a mouse? A touchpad? Or steps on a keyboard? Please specify. Also, are “-144 to 144” arbitrary units or pixels? If they are arbitrary units, I think it is confusing to mention them. Did each participant watch all the segments of a movie, or just some? Are the trials self-paced? More importantly, what was the task instruction? Different terms used in questions lead to different results in aesthetic studies.
(5) In “Behavioral Data Processing”, it is not clear how " cuts " were quantitatively defined. Measuring latency by “first rating change following each cut” assumed that there was no rating change that corresponded with the end of the previous cut, which sounds dubious. Please justify this assumption.
(6) In “Color Statistics”, if colorfulness was computed from descriptive statistics of greenness and blueness, why were they all included in the later analysis? It seems redundant. What additional information do greenness and blueness tell us?
(7) In “Color Statistics,” again, the quantitative definition of cue contrast is unclear. How do colors “spread out” if hues are on a circular scale? Based on Figure 2 and the narratives, I think the authors meant saturation contrast.
(8) In “Color Statistics”, brightness contrast was computed in a peculiar way. The computation is not evident in the provided citation. Why not use a standard contrast measure, e.g., RMS contrast?

References

Graf, L. K., & Landwehr, J. R. (2015). A dual-process perspective on fluency-based aesthetics: The pleasure-interest model of aesthetic liking. Personality and Social Psychology Review, 19(4), 395-410. https://doi.org/10.1177/1088868315574978

Redies, C. (2015). Combining universal beauty and cultural context in a unifying model of visual aesthetic experience. Frontiers in human neuroscience, 9, 218. https://doi.org/10.3389/fnhum.2015.00218

Version 1:

Reviewer comments:

Reviewer #1

(Remarks to the Author)
The authors have addressed my comments well. The revised manuscript is much improved.

Reviewer #2

(Remarks to the Author)
I am happy to see that all my concerns were addressed. I appreciate the authors' responses. One additional comment is that the dragging finger in Figure 1 could be misleading given that the participants responded by arrow keys.

Open Access This Peer Review File is licensed under a Creative Commons Attribution 4.0 International License, which permits use, sharing, adaptation, distribution and reproduction in any medium or format, as long as you give appropriate credit to the original author(s) and the source, provide a link to the Creative Commons license, and indicate if changes were made.
In cases where reviewers are anonymous, credit should be given to 'Anonymous Referee' and the source.
The images or other third party material in this Peer Review File are included in the article’s Creative Commons license, unless indicated otherwise in a credit line to the material. If material is not included in the article’s Creative Commons license and your intended use is not permitted by statutory regulation or exceeds the permitted use, you will need to obtain permission directly from the copyright holder.
To view a copy of this license, visit https://creativecommons.org/licenses/by/4.0/

Response to Reviewer Comments

We thank the two reviewers for their constructive comments on our manuscript. Following their comments, we have thoroughly revised our manuscript. Detailed responses to the reviewer comments can be found below (in blue), together with the key changes made to the manuscript (in red).

Reviewer 1:

The authors of this manuscript endeavor to identify the visual features underlying the aesthetic evaluation of movies. The presented participants with two quite different movies while participants were asked to continuously rate their aesthetic enjoyment. The authors then analyze the feature content of the movies in feature groups of "visual fluency", "symmetry", "color statistics", and "visual motion". Using Ridge Regression, they then predict participants' aesthetics ratings both within and across subjects, and both within and across movies. They find color statistics to be the most influential feature for predicting aesthetic liking.

The study is well designed, the analysis is well motivated, and the paper is well written. I have a few comments and questions.

We thank the reviewer for the overall positive assessment. We respond to their remaining questions below.

1. My main issue with the manuscript is that this seems fairly thin data on which to stake bold claims. The two movies shown here are quite different, but each is also very specific in content and visual features. How can we know that the main claim of the paper – that color statistics explain dynamic aesthetic experiences – holds in general? Yes, the main finding replicates across data sets. In the end, I believe that the authors can really only make claims about those two particular movies. The authors are cautious not to overstate their results. Yet, the stimulus-specificity of the results limits the broader impact of the findings.

We thank the reviewer for this comment. As our study is based on two specific movies, we indeed cannot know whether the same features would generalize to other movies. Here, we aimed to test whether a combination of different visual features can predict aesthetic judgements to two movies that are markedly different from each other in both content and visual style. We agree that broader generalization requires additional datasets spanning a wider range of genres and visual styles. We revised the manuscript to avoid strong claims regarding generalizability and highlight this issue in the newly added "Limitations" section (Page 16).

"Two limitations of our study warrant further attention. First, our findings are based on only two movies. Although these movies differ a lot in both content and visual style, they cannot capture the full diversity of naturalistic dynamic visual experiences. Our findings should be interpreted as evidence that visual-feature models generalize two very distinct movies and across viewers, rather than as demonstrating universal feature preferences. Future work needs to examine a wider range of movies, genres, and visual contents to determine the extent to which these feature preferences generalize across naturalistic contexts."

We revised the paragraph on "**How "good" are models based on interpretable visual features?"** at page 16:

"Finally, it is important to emphasize that these findings are based on two visually and semantically distinct movies. Although successful predictions across these two distinct movies support the generalizability of our findings to some extent, a broader range of visual styles and semantic content is necessary to establish the generality of the identified feature–aesthetic relationships and determine whether color statistics play a dominant role in dynamic aesthetic experiences more broadly."

We also rewrote the conclusion at page 17:

"In sum, our findings suggest that aesthetic experience during movie watching is partly shaped by shared perceptual mechanisms captured by visual features, while individual differences and contextual factors continue to play an important role. Across two visually distinct movies, these feature-based relationships generalized across observers and movie content, highlighting the contribution of perceptual processing to dynamic aesthetic experience. Future research should include a broader range of visual content and integrate low-level feature-based approaches with higher-level cognitive and affective factors to develop a more comprehensive model of aesthetic experience."

2. The authors correctly emphasize the differences in visual features between the two movies (e.g., in the 5th paragraph of the introduction, at the bottom of page 2). I strongly recommend that they should also comment on the differences in content, which will likely affect aesthetics ratings to a large degree.

This is a great suggestion. We included additional information on this, at the bottom of page 2:

"Whereas *Home* is a nature documentary about the Earth and humanity's relationship with its natural resources, depicting diverse real-world landscapes and environmental scenes with varying levels of aesthetic appeal, *Loving Vincent* is an animated biographical film that retells the life of Vincent van Gogh through a highly stylized painterly aesthetic. Thus, the two movies differ substantially not only in their visual appearance but also in their semantic content, narrative structure, and emotional themes."

We focus more strongly on the visual differences for the remaining manuscript because our models are mostly designed to capture image-computable low- to mid-level visual features, as noted in "**How "good" are models based on interpretable visual features?"** at page 15:

"In particular, our models focused on image-computable visual features of low to intermediate complexity and therefore did not explicitly capture differences in semantic content or narrative structure, which likely also contribute to dynamic aesthetic experiences."

3. Page 9, second paragraph: The authors observe an asymmetry in the variability of cross-movie predictions. Their explanation (sentence starting with "This may reflect …") is a bit nebulous. I would encourage the authors to explore this issue further, ideally with additional analyses of the properties of the data sets. I believe that the asymmetry is a significant finding that deserves a bit more attention and interpretation.

Thanks for this suggestion. To be completely transparent, we currently do not have a good explanation for this asymmetry. In the manuscript, we suggested that *Loving Vincent* occupies a narrower visual feature space, and that's why models trained on this movie exhibited lower prediction variability. To test this hypothesis, we conducted a principal component analysis of the extracted visual predictors for *Home* and *Loving Vincent*, which is reported on page 11:

"This asymmetry may reflect differences in the structure of the visual feature space across the two movies. Specifically, *Loving Vincent* occupies a lower-dimensional feature space than *Home*, meaning that its visual content can be described by fewer independent combinations of visual features. Indeed, a principal component analysis of the extracted visual predictors supported this interpretation: the first principal component explained a larger proportion of the variance in *Loving Vincent* (40.8%) than in *Home* (23.9%), and only six principal components were required to explain 90% of the variance, compared with eight for *Home* (Fig. S5)."

We also added the following figure to our "Supporting Information" document:

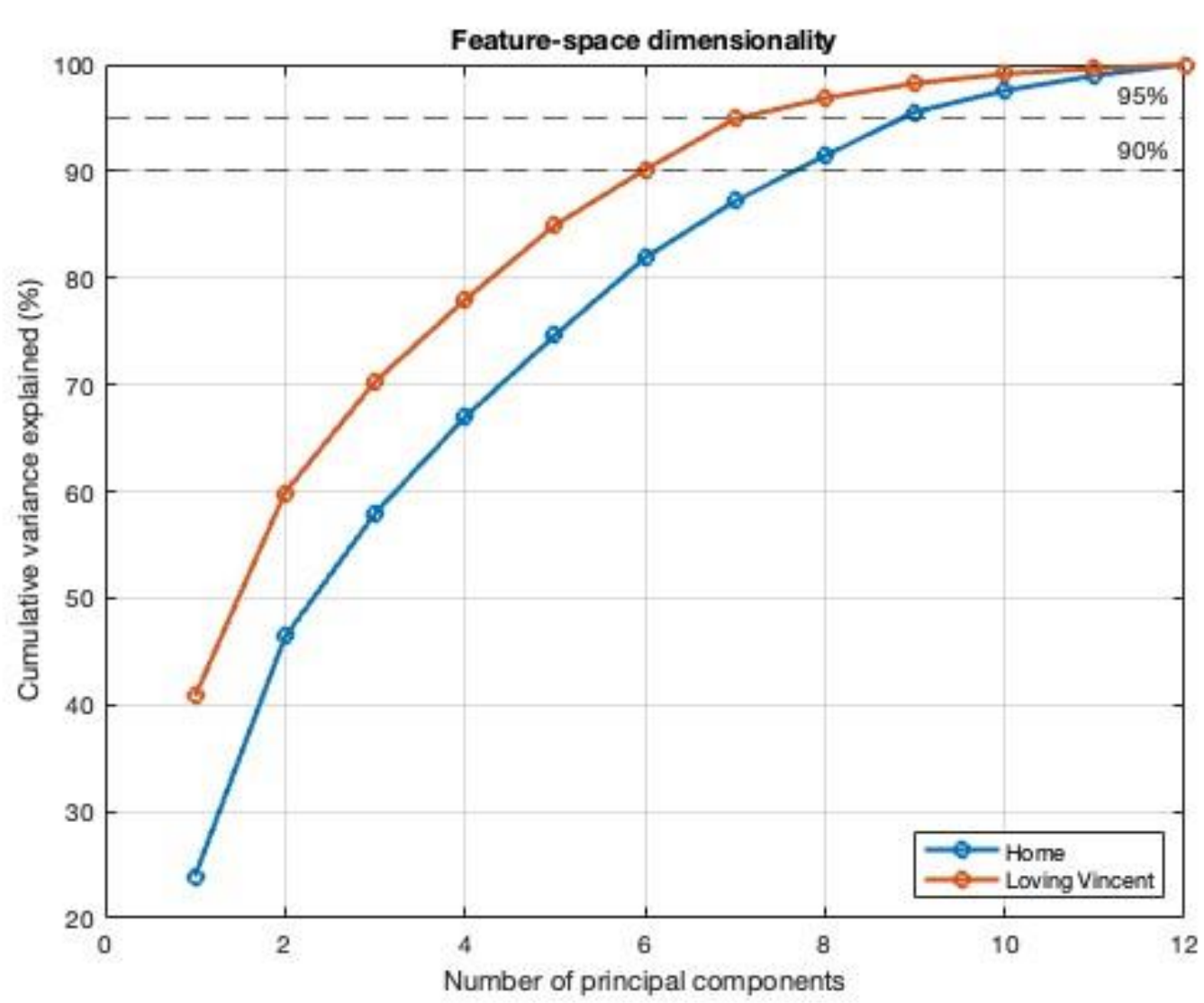

Figure S5. **Principal component analysis of the visual feature space.** Cumulative variance explained by successive principal components for the 12 visual predictors extracted from *Home* and *Loving Vincent*. *Loving Vincent* exhibited a lower-dimensional feature space than *Home*.

4. Page 12, 3rd paragraph (discussion of color): Why did black-and-white movies ever capture the audience's attention? Why should green and blue be particularly important for aesthetic appeal? Do these hues correlate with particular content, such as landscapes, which are known to be aesthetically preferred across genres? I am looking for insights into aesthetics here. The way the discussion is written, the authors could have commented equally well on any other of the features to turn out to be important. What is it about color? Is it its relationship to emotions? Here is where the important scientific work needs to happen.

That is a great question. Our data suggests that in *Loving Vincent*, which includes both colorful and black-and-white scenes, observers prefer color over black-and-white, as reported in the revised manuscript on "**Color predicts aesthetic appeal across different content**" at page 14:

"Notably, our finding that color was an important predictor across two movies should not be interpreted as implying that color is necessary for aesthetic experience. Rather, our findings suggest that, when color information is present, it provides particularly informative cues for predicting dynamic aesthetic preferences. Supporting this interpretation, an exploratory analysis of *Loving Vincent*, which contains both colorful and (largely) monochromatic scenes (40.8% of the movie), showed that scenes with minimal color information received significantly lower aesthetic ratings than colorful scenes ($t(29) = -2.28$, $p = .03$, Cohen's $d = -0.42$, $CI = [-0.8, -0.04]$). Thus, when chromatic and achromatic scenes coexist within the same movie, chromatic scenes elicit significantly higher aesthetic ratings, further supporting the contribution of color to aesthetic experience. However, in the absence of color, as in Charlie Chaplin films or Magnum Photos, other visual features, such as luminance, texture, and motion, as well as higher-level factors including narrative meaning and emotional engagement, may play a greater role in shaping aesthetic appeal."

While this may suggest that black-and-white movies are liked less, this evidence should rather be taken as anecdotal. To further dive into why color might be important, we revised the paragraph on "**Color predicts aesthetic appeal across different content**" at page 13:

"Particularly, color features were the only predictors that robustly generalized across movies. Among the color predictors we used, overall variation in colors (colorfulness), along with brightness contrast, contributed most to successful predictions of aesthetic ratings (Fig. S3), consistent with prior work showing that colorfulness is a significant predictor of aesthetic preference [48]. The positive contribution of colorfulness (positive beta coefficient) indicates a preference for colorful visual inputs. One possible explanation is that color conveys not only low-level visual information but also higher-level ecological and emotional cues. According to the Ecological Valence Theory (EVT) of color preference, preferences for specific colors arise because they are associated with positively or negatively valued objects and environments rather than because of the colors themselves [37]. Moreover, our results indicate that green and blue hues are associated with greater aesthetic appeal than red and yellow hues (Fig. S3). Consistent with this pattern, earlier work has shown that bluish and greenish color ranges tend to be preferred over darker or brownish tones in aesthetic evaluations of paintings [49]. According to the EVT, the positive contributions of green and blue hues in our models may reflect the positive affective associations that these colors acquire through experience with natural environments

[37]. Specifically, blue and green are commonly associated with aesthetically preferred natural elements such as clear skies, water, and rich forests. Thus, rather than reflecting an isolated preference for these hues alone, their contribution to aesthetic appeal may partly arise from the positive ecological and semantic information they convey. Color may therefore play an efficient role that more broadly captures perceptual properties of a scene and aspects of its semantic and emotional content, potentially explaining why color-related features generalized more robustly across two visually and semantically distinct movies than the other visual features considered here."

5. By comparison, fluency did not predict aesthetic preference well across movies (page 13, second paragraph). The authors go to great lengths to explain why this might be the case by invoking differences in movie content and emotionality. Why would these differences matter for fluency but not for other features? Emotions are strongly tied to colors, so why do these differences not appear in the color analysis?

This is an interesting question. According to the Ecological Valence Theory (EVT), colors acquire emotional meaning through association with objects and environments that people perceive positively or negatively, rather than from the colors themselves (Palmer & Schloss, 2010). Therefore, these learned associations may remain relatively stable across different visual contexts and artistic styles, allowing color statistics to retain a more consistent relationship with aesthetic preference across stimulus domains. Moreover, our color predictors describe relatively low-level image properties that are computed in the same way across naturalistic and artistic images. In contrast, the fluency measures were derived from higher-order representations of a deep neural network (VGG16) trained on natural scene categorization. The categorization objective may yield differential predictions for the differing contents across the two movies.

6. I believe that point 5 above illustrates the issue that I raised in point 1. If the interpretation of the results requires detailed discussion of the idiosyncrasies of each of the movies, then perhaps two movies aren't enough to make general statements. It's still a fine paper when the story is about these particular two movies, but its impact is not the same as if the authors could make more general claims.

We fully agree with this point. We have made several changes to highlight that general claims require further studies with more diverse stimulus sets (see our response to point 1).

7. One minor methods detail: Page 16, 5th paragraph: The movies are divided into 8x8 squares. Does that mean that the movies are cropped to a square for the experiment? If so, then this should be mentioned and justified.

During the experiment, the movies were shown at their native resolution and aspect ratio (1280x720 pixels). For computing the fluency predictors, movie frames were divided into 8x8 grids of equal-sized squares. As the VGG16 we used for this purpose demands square inputs, we forced each frame into a square. This is now mentioned in the methods at page 6:

"We presented the network with either the full movie frame or spatially partitioned versions of the frame. Because the VGG16 network we used requires square image inputs, each movie frame was resized from its original resolution (1280 × 720 pixels) to 640 × 640 pixels**.** The partitions were then generated by dividing each resized frame into an 8 × 8 grid of equal-sized squares. Two complementary image sets were created: one containing all odd-numbered squares and the other containing all even-numbered squares, analogous to the black and white fields of a checkerboard pattern. The full image and both partitioned versions were fed separately into the network, and layer-specific activation patterns were extracted from the 11th layer, as previous work indicated that this layer yields the most robust predictions of aesthetic appeal [33]."

Reviewer 2:

This study investigated how aesthetic appeal is perceived from videos. Participants continuously reported aesthetic appeal while watching movie clips, and their responses were compared with computational metrics describing video characteristics. The study made a progressive attempt to investigate aesthetic appeal along time series instead of static displays. It is interesting to see that the predictions generalized between two types of movies and across participants.

We thank the reviewer for the overall positive assessment. We respond to their remaining questions below.

The fact that this study investigates time series leads to the first concern. The current analysis of human responses is at risk of losing substantial information and becoming misleading. To start with, the granularity of the recorded data is unclear. Participants continuously responded using a sliding bar. Although the responses were described as "moment-to-moment", I highly doubt that analog signals were recorded. What was the sampling rate of human responses? A similar issue applies to the stimuli. The authors only indirectly mentioned that the videos had a frame rate of 25 fps and did not specify whether the videos were displayed at the same frame rate via Psychtoolbox.

We are happy to clarify these points. First, movies were presented frame-by-frame at their native frame rate (25 frames/s) using Psychtoolbox. This is now mentioned in "Procedure" section at page 5:

"Stimuli were displayed on a monitor with a resolution of 1280 × 720 pixels and a refresh rate of 100 Hz. Movie frames were presented sequentially at their native frame rate of 25 frames/s using Psychtoolbox. At this viewing distance, the stimuli subtended 28.7 × 16.4 degrees of visual angle."

Second, the rating task did not record an analog input device (e.g. joystick or mouse), but was operated by button presses. The current slider position was sampled once per video frame (25 Hz). Between adjustments, the slider position remained constant. This is mentioned in "Procedure" section at page 5:

"Participants adjusted the slider using the right or left arrow keys. The position of the rating

slider was sampled once per movie frame (25 Hz). Between button presses, the slider position remained constant, resulting in a frame-by-frame record of participants' continuous ratings"

The hazard lies in the following procedure. Although not overtly specified, I presume that human responses were binned by one-second slots in congruence with computational metrics, "because faster changes would probably not be reflected prominently in an adjustment of the rating slider." This claim might be true, but it needs to be supported by empirical evidence instead of speculation. A sampling rate of 1Hz leads to a Nyquist frequency of 0.5Hz, which is one oscillation every 2 seconds. I can easily imagine participants moving the sliding bar at a higher frequency, and a sampling rate as low as 1Hz is likely to induce aliasing, which is an artifact that causes misrepresentation. To better represent human responses, the authors need to determine a range of relevant frequencies based on the raw data of human responses (which were presumably sampled at an excessively high rate), and bin their data by a time slot corresponding to twice the highest relevant frequency (see Nyquist–Shannon sampling theorem). The resulting time slot might end up being close to one second, but I would like to see evidence of this choice.

This is an important point. To empirically justify the temporal resolution used in our analyses, we analyzed the power spectrum of the raw framewise rating traces (25 Hz) prior to temporal averaging. Using Welch's power spectral density estimate, we determined the frequency below which 95% and 99% of the cumulative signal power was contained.

For Experiment 1, the median frequencies containing 95% and 99% of the signal power were 0.049 Hz and 0.146 Hz, both well below the 0.5 Hz Nyquist frequency associated with a 1-Hz sampling rate. For Experiment 2, the corresponding values were 0.073 Hz and 0.220 Hz, again well below the 0.5 Hz Nyquist frequency. These results indicate that the temporal dynamics of the behavioral ratings were substantially slower than the frequency limit imposed by the 1-s binning procedure, suggesting that the aggregation preserved the relevant temporal information with minimal risk of aliasing.

To be sure that analyzing the data with a 1-second resolution did not corrupt the analysis, we reran the main ridge regression for both movies at the native frame-to-frame resolution of 25 Hz. As highlighted in the figure below, the results were virtually identical. We thus decided to keep the analysis at a 1-second resolution in the manuscript.

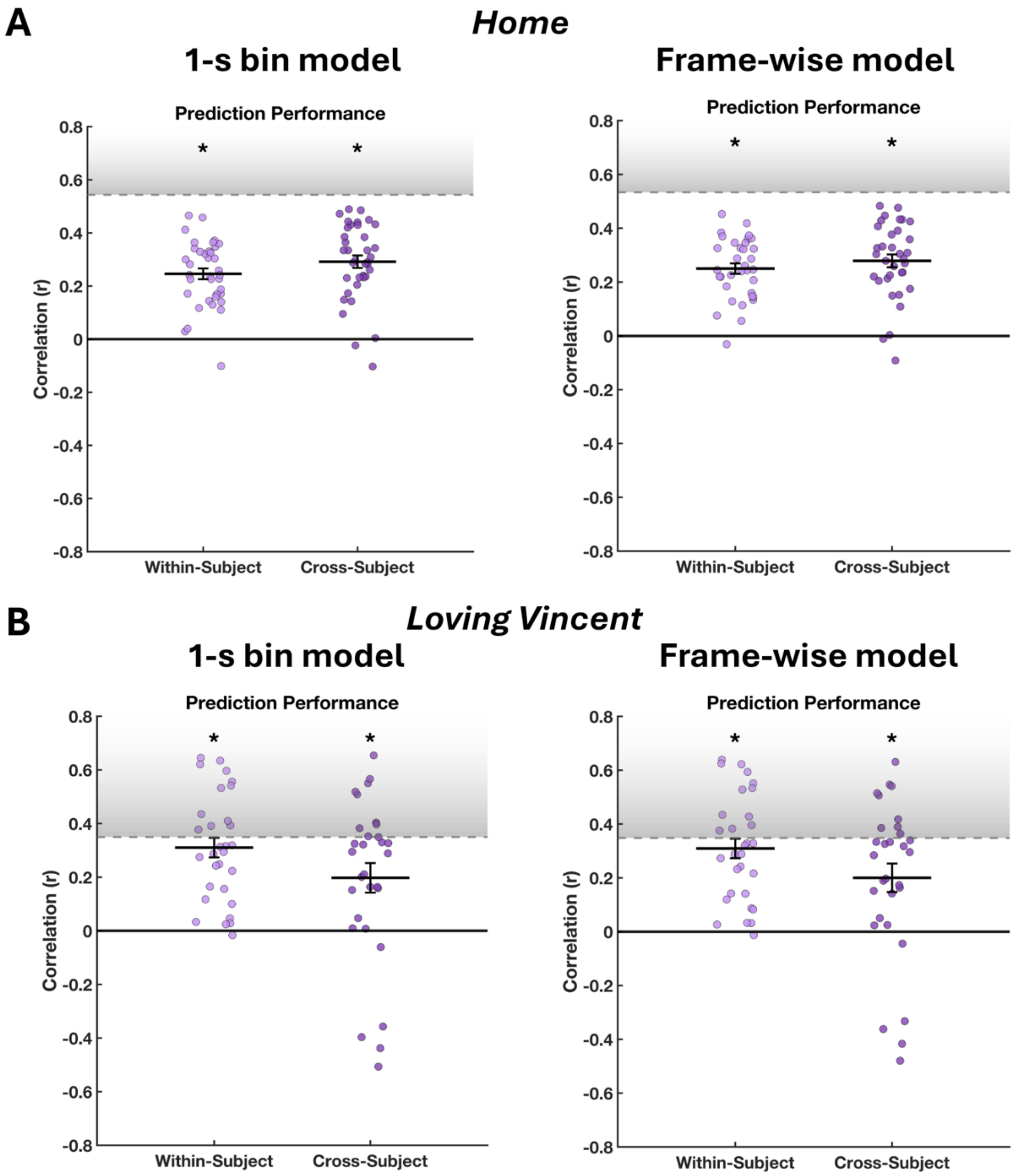

Caption: Within- and cross-subject prediction performance for *Home* (A) and *Loving Vincent* (B). Prediction performance is compared between models using 1-second binned ratings and predictors (1 Hz) (shown on the right) and frame-wise ratings and predictors (25 Hz) (shown on the left). The two temporal resolutions yielded comparable prediction performance, with no notable differences between the models. Error bars represent SEM. Asterisks indicate $p < 0.05$.

Finally, it is worth noting that different degrees of temporal shifting between the rating data relative to the visual feature predictors did not drastically impact the results (see our response to point (5) below), suggesting that fast fluctuations in the responses play a less critical role.

The second concern is also related to time series analysis. I believe that the authors binned computational metrics and human responses by one-second slots and treated each slot as an

independent data point in the regression. This analysis diminishes temporal dependency, which is a crucial difference between videos and static displays. Also, the computational metrics were computed independently for each frame, i.e., information in stimuli was integrated spatially but not temporally. I speculate that the predictability of a given frame from previous frames would correlate with aesthetic appeal.

We agree that analysis of the temporal evolution of different features, including their predictability over time, would be very interesting. However, our movies change rather slowly across time and thus are probably not ideal for performing such an analysis.

To quantify this intuition, we computed the pixelwise correlation between grayscale movie frames separated by symmetric temporal windows around each time point. Specifically, for each lag L, we correlated the frame at $t - L$ with the frame at $t + L$, corresponding to a total temporal separation of $2L$ frames. We repeated this analysis for lags ranging from 1 to 25 frames (0.08–2 s total separation), thereby characterizing how visual similarity decreased with increasing temporal distance. Windows spanning automatically detected shot boundaries were excluded to avoid artificially low correlations introduced by scene cuts.

In Experiment 1, across all movie segments (113,654 movie frames), for the temporal separation corresponding approximately to our 1-s behavioral aggregation window (25 frames), the median pixelwise correlation was 0.738 (mean = 0.683) after excluding windows crossing detected shot boundaries. These results indicate that neighboring one-second intervals generally contained highly similar visual information, supporting the use of 1-s temporal aggregation while preserving the inherent temporal continuity of the movie stimuli. Similarly, in Experiment 2, across all movie segments (116,257 movie frames), the median pixelwise correlation within the 1-s behavioral aggregation window (25 frames) was 0.870 (mean = 0.786) after excluding windows crossing detected shot boundaries.

Generally, we agree that studying temporal variations in visual features is an interesting avenue for future studies. However, these studies will need to employ (i) more changeable visual stimulation, and (ii) more temporally fine-grained metrics for assessing visual preferences.

The third concern is that the authors made too strong a claim on the contribution of perceptual features to aesthetic appeal ("aesthetic experience is best understood as shared subjectivity"). The correlations between model predictions and observations are not strong (smaller than 0.4 in Figure 3 and smaller than 0.2 in Figure 5). Moreover, the current paradigm enhances the effects of perceptual factors. Compared with a common design that requires participants to respond after exposure to stimuli, the current design requires participants to complete two tasks simultaneously: watching the video and responding. The latter task divides attention. The PIA model (Graf & Landwehr, 2015) separates aesthetic processing into automatic and controlled processes and specifies that attention is required to enter the controlled process. Dividing attention inhibits the contribution of the controlled process, which involves "cognitive enrichment". The same prediction can be made from another theoretical framework. Redies (2015) distinguished between the aesthetics of perception and the aesthetics of cognition. Simultaneous reports raise cognitive load, which inhibits the aesthetics of cognition and increases the contribution of the aesthetics of perception.

We thank the reviewer for this comment. We agree that our original wording overstated the contribution of perceptual features to aesthetic experience.

Our results show that perceptual features explain an important component of dynamic aesthetic experiences, rather than aesthetic experience in its entirety. Although the prediction accuracies are moderate, as the reviewer correctly says, the maximally attainable correlations (given inter-subject agreement) are 0.54 and 0.35 for Experiments 1 and 2, respectively. Thus, while our models do not account for the full range of factors underlying aesthetic experience, they explain a substantial proportion of the explainable variance. Yet, there is clearly additional variance to explain.

It is possible that continuous rating increases the contribution of perceptual features to aesthetic experience, given that continuous ratings put additional strain on cognitive processing. However, empirical work from Isik & Vessel (2019) showed that participants who continuously rated their preference for natural videos did not differ in their overall ratings or inter-observer agreement from participants who viewed the videos passively and rated them afterward. This suggests that continuous reporting can capture temporal fluctuations in aesthetic experience without substantially altering the overall aesthetic evaluation. We have added this point to the Discussion/Limitations while still acknowledging that continuous reporting may relatively emphasize perceptual processing, see page 16:

"Second, the continuous rating paradigm used here may have emphasized perceptual processing, with concurrent movie watching and rating reports putting additional strain on cognitive resources. This may tip the balance between rapid, automatic perceptual processing and slower, controlled cognitive processing involved in aesthetic appreciation [8,62]. While we cannot exclude that this tipped balance leads to an overestimation of perceptual factors, the differences may not be so pronounced in practice: As Isik & Vessel (2019) reported previously, preference judgments for short movies do not differ systematically between continuous "online" ratings and "offline" ratings after the movie presentation."

(1) In Figure 1, I am not sure what the tiny rating bars in the line plots stand for, and they do not seem to correspond with the line plot values. Please remove or specify.

Tiny rating bars intended to represent how the continuous rating changes over time. We realized this could be confusing and decided to remove them.

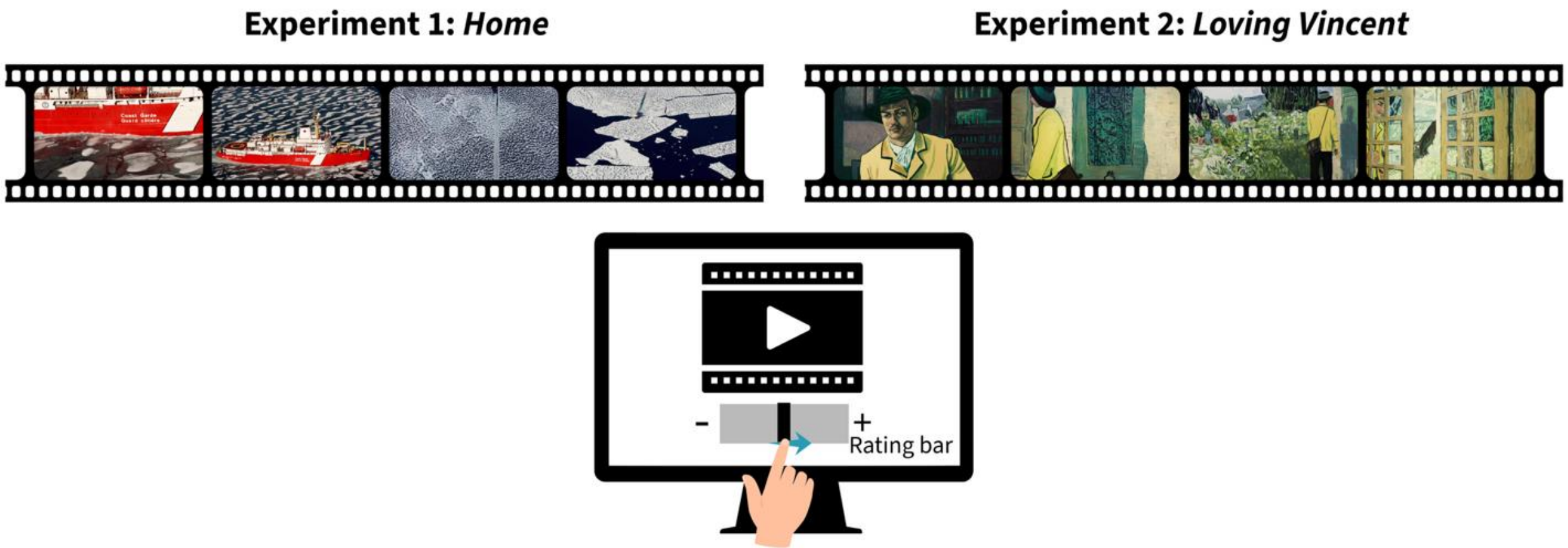

Figure 1. Experiment paradigm**.** Participants continuously rated the aesthetic appeal of the movies *Home* (Experiment 1) or *Loving Vincent* (Experiment 2) using a slider bar.

(2) Please include a gross introduction on computational metrics in the introduction section, i.e., mentioning the contents in Figure 2. It is not clear what the "multiple visual properties" are and why they are important/relevant.

We added a new paragraph at the top of page 3 explaining our computable visual features and why we selected them specifically:

"For both movies, we extracted twelve image-computable features belonging to four broad categories: visual fluency, symmetry, color statistics, and motion energy (Fig. 2). These features were selected because previous empirical and computational studies have identified them as important predictors of aesthetic preference. Specifically, visual fluency, symmetry, and color statistics have each been associated with aesthetic judgments across a variety of static visual stimuli, whereas motion energy was included to examine whether dynamic visual information provides an additional contribution to aesthetic experiences during movie viewing. The visual fluency category included three predictors, similarity, integration, and L2 norm, derived from deep neural network representations. These measures have previously been shown to predict aesthetic preferences across multiple datasets of naturalistic images [33]. They capture mid-level spatial organization of an image by quantifying how efficiently different parts of an image are integrated within a visual representation, allowing us to test whether deep neural network-based measures of visual fluency also explain dynamic aesthetic experiences during movie viewing. Symmetry has consistently been linked to aesthetic preference across a wide range of visual stimuli [34,35]. Accordingly, we included computational measures of both vertical and horizontal symmetry to examine their contribution to dynamic aesthetic experiences [36]. Lastly, we computed a broad range of color statistics to capture different aspects of color perception. Color is a unique visual feature because it conveys both low-level perceptual information and higher-level emotional and semantic associations, making it an important determinant of

aesthetic preference [37,38]. To build on computational measures that have previously been shown in aesthetics research, we included colorfulness, greenness, and blueness [39], as well as hue contrast, brightness contrast, and mean saturation [4,40].”

(3) In “Movie Stimulus”, why were there 8 randomized sequences in Experiment 1 and 18 in Experiment 2? The number of segments between the two movies was roughly the same. Please explain.

Thanks for pointing this out. When looking into this, we realized that we made an error in the initialization of the random sequences in Matlab, where we did not appropriately set random seeds for each participant. The final dataset thus only contained 8 unique sequences in Experiment 1 and 18 unique sequences in Experiment 2. We do not think that this error has any systematic influence on the results, which would be equally interesting with a single, fixed sequence. We now point out this problem in the methods section at page 4:

“Movie segments were presented in randomized order, and there were 8 unique presentation sequences in Experiment 1 and 18 unique presentation sequences in Experiment 2. The (differing) number of random sequences was related to an error in implementing the randomization”

(4) In “Procedure”, did the participants respond by dragging a bar with a mouse? A touchpad? Or steps on a keyboard? Please specify. Also, are “-144 to 144” arbitrary units or pixels? If they are arbitrary units, I think it is confusing to mention them.

Participants continuously rated their aesthetic experience using the left and right arrow keys on a keyboard. The rating slider was displayed at the bottom of the screen and ranged from −144 to 144 pixels. Each key press moved the slider by 6 pixels, resulting in 49 discrete rating positions. The center of the slider indicated a neutral rating, whereas the right represented higher and the left represented lower aesthetic experience.

Did each participant watch all the segments of a movie, or just some? Are the trials self-paced? More importantly, what was the task instruction? Different terms used in questions lead to different results in aesthetic studies.

Each participant viewed all movie segments of fixed duration while continuously adjusting their ratings. They could move the slider at any time, but they could not control when a trial (movie segment) ended. We updated the procedure description at page 5 to include further information on the exact instructions:

“In both experiments, participants sat in a darkened room with their heads stabilized by a chin and forehead rest, positioned 68 cm from the screen. Stimuli were displayed on a monitor with a resolution of 1280 × 720 pixels and a refresh rate of 100 Hz. At this viewing distance, the stimuli subtended 28.7 × 16.4 degrees of visual angle. A practice session of continuous aesthetic rating was shown at the beginning of each experiment. The experiment was programmed using

Psychtoolbox version 3.0.19 in MATLAB R2022a (MathWorks, Natick, MA, USA) on a Windows 10 PC. During the movie segments, each participant watched all segments and rated aesthetic appeal using a slider presented below the movie. Participants adjusted the slider using the right or left arrow keys. If participants did not provide a response for more than 5 seconds, the slider flickered red and green. In this case, they could press the up arrow to indicate that their rating was still the same. The text shown in the on-screen instruction was as follows: *"While watching the video, please continuously rate your aesthetic experience using the LEFT and RIGHT ARROW keys. The rating bar will appear at the bottom of the screen. The center of the bar represents a neutral rating. The right side represents higher aesthetic experience, and the left side represents lower aesthetic experience. If you do not respond within 5 seconds, the rating bar will flicker red and green. While flickering, if your aesthetic experience stays stable, you can press the UP ARROW.""*

(5) In "Behavioral Data Processing", it is not clear how " cuts " were quantitatively defined. Measuring latency by "first rating change following each cut" assumed that there was no rating change that corresponded with the end of the previous cut, which sounds dubious. Please justify this assumption.

We apologize for not describing the cut detection procedure in sufficient detail. We revised "Behavioral data processing" section in methods at page 5:

"Cuts were identified from framewise visual changes. For each pair of consecutive frames, both frames were converted to grayscale and the mean pixel-intensity difference across all pixels was computed. Frame transitions exceeding predefined positive or negative thresholds were identified as candidate cuts, and consecutive suprathreshold frames were grouped into a single cut region. The first frame of each cut was used as the temporal reference point for the latency analysis. Thresholds were decided by trying range of different values and visually inspecting the resulting cuts for both movies."

We agree that overlapping cuts could potentially confound the estimation of response latency. To address this concern, we performed an additional robustness analysis in which we shifted the ratings by fixed delays ranging from 0 to 5 s in 0.5-s increments and compared the resulting prediction performance with that obtained using our individually estimated response delays. The results for both movies showed that prediction performance was largely stable across the range of fixed delays. Moreover, using individually estimated response delays yielded slightly better prediction performance than any of the fixed-delay approaches. The mean estimated response delay in the Experiment 1 was 62.1 frames (*Home*) and in the Experiment 2, it was 55.2 (*Loving Vincent*). There is also a small peak in the middle of delay time ranges around 2 seconds (50 frames), which is close to our mean estimated delay times (Figure S6). We also added the following figure to our "Supporting Information" document and added the following text in the Methods at page 5:
"The mean estimated latency before applying the two-second cap was 62.1 frames in Experiment 1 (*Home*) and 55.2 frames in Experiment 2 (*Loving Vincent*). As a robustness check, we also evaluated fixed temporal shifts ranging from 0 to 5 s in 0.5-s increments and compared the resulting prediction performance to that obtained with individually estimated delays (Fig. S6)."

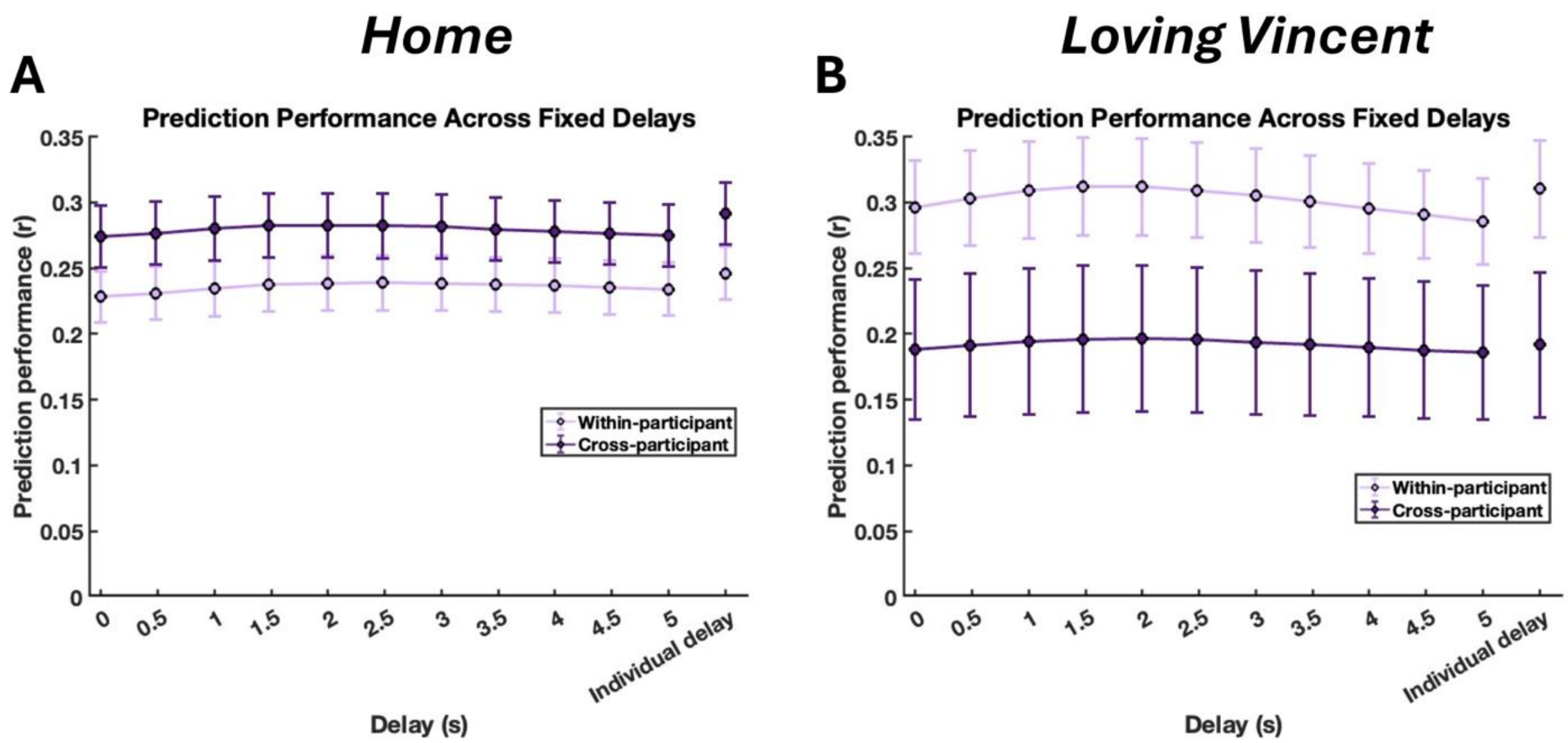

Figure S6. **Robustness of prediction performance across fixed response delays.** Prediction performance was evaluated after shifting the behavioral ratings by fixed delays ranging from 0 to 5 s in 0.5-s increments. Results are shown separately for *Home* (A) and *Loving Vincent* (B). Prediction performance remained largely stable across the tested delays, while the individually estimated response delays yielded slightly better performance than the fixed-delay approaches.

(6) In "Color Statistics", if colorfulness was computed from descriptive statistics of greenness and blueness, why were they all included in the later analysis? It seems redundant. What additional information do greenness and blueness tell us?

Even though the colorfulness measure is computed from both the mean and standard deviation of the CIELab *a* and *b* channels, it measures only the overall magnitude of chromatic variation, not the direction. Because it is based on the Euclidean norm, it does not distinguish whether the dominant colors are green or red, or blue or yellow. For example, an image with strong green colors can have an identical colorfulness score to a red dominant image, but they are opposite in greenness. The same logic applies to blue and yellow dominant images. As also mentioned in the paper, Nakauchi et al. (2021) found that people prefer greenish and bluish color ranges over darker or brownish colors in their evaluations of paintings. Therefore, greenness and blueness preserve this directional information and provide complementary information beyond colorfulness.

(7) In "Color Statistics," again, the quantitative definition of cue contrast is unclear. How do colors "spread out" if hues are on a circular scale? Based on Figure 2 and the narratives, I think the authors meant saturation contrast.

We thank the reviewer for pointing this out. Following Iiagaya et al. (2021), hue contrast was computed from the angular distances between dominant hue bins on the circular hue histogram after excluding pixels with low saturation or extreme brightness. The measure shows the angular span of the dominant hues on the hue circle. When the largest gap between dominant hue bins

exceeded π, we used the smaller angular distance (2π − gap) to account for the circular nature of hue. We also updated our color statistics section on page 7:

"Hue contrast was computed following Iiagaya et al. (2021). Pixels with low saturation or extreme brightness were excluded, and the remaining pixels were assigned to bins on a circular hue histogram. Hue contrast was defined as the angular span of the dominant hue bins on the hue circle. Because hue is a circular variable, if the largest angular gap between dominant hue bins exceeded π, the complementary angular distance (2π − gap) was used. Larger values indicate a broader range of dominant hues in the image."

(8) In "Color Statistics", brightness contrast was computed in a peculiar way. The computation is not evident in the provided citation. Why not use a standard contrast measure, e.g., RMS contrast?

Brightness contrast was computed following the implementation of Iiagaya et al. (2021), based on the measure originally proposed by Li and Chen (2009). We included this measure because Iiagaya et al. (2021) demonstrated that it successfully predicted aesthetic ratings. As our goal was to evaluate image features that have previously been linked to aesthetic preference, we adopted their definition of brightness contrast rather than replacing it with a standard measure such as RMS contrast. Specifically, brightness contrast was defined as the width of the narrowest brightness range centered on the modal brightness value that contains 98% of the image pixels.

Response to Reviewer Comments

We thank the two reviewers for their constructive comments on our manuscript. Following their comments, we have thoroughly revised our manuscript. Detailed responses to the reviewer comments can be found below (in blue), together with the key changes made to the manuscript (in red).

Reviewer 1:
The authors have addressed my comments well. The revised manuscript is much improved.

We thank the reviewer.

Reviewer 2:
I am happy to see that all my concerns were addressed. I appreciate the authors' responses. One additional comment is that the dragging finger in Figure 1 could be misleading given that the participants responded by arrow keys.

We thank the reviewer. We removed the dragging finger from Figure 1.

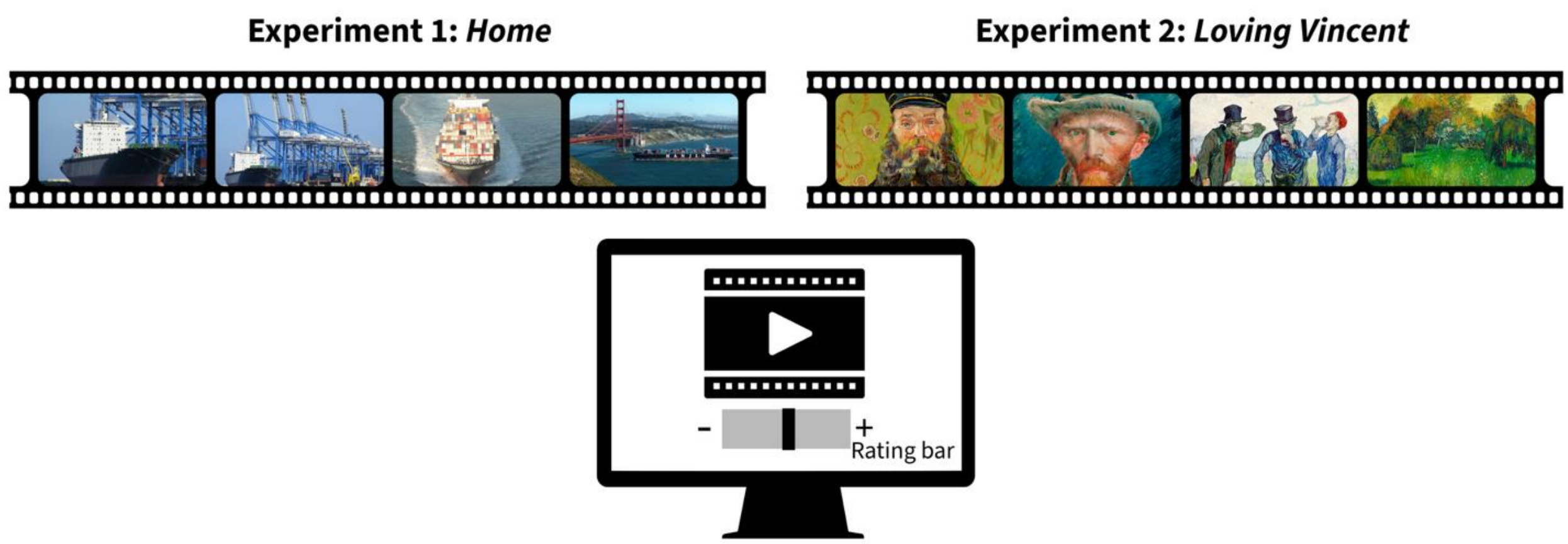

Figure 1. Experiment paradigm. Participants continuously rated the aesthetic appeal of the movies *Home* (Experiment 1) or *Loving Vincent* (Experiment 2) using a slider bar. Images shown are illustrative and were not used as experimental stimuli. The original experimental stimuli were the movies *Home and Loving Vincent.* Images are licensed under CC BY 4.0. Photographers, in order: Sludge G, Daniel Ramirez, John Morgen, Free Public Domain Illustrations. Images were obtained through Openverse.
