# Supplementary material for: Visual features explain dynamic aesthetic experiences across distinct movie content
Source: Commun Psychol. 2026 Sep 11;4:127. doi: 10.1038/s44271-026-00531-7 (PMC13569840; doi:10.1038/s44271-026-00531-7)
Supplement: Supplementary file 2 [file 44271_2026_531_MOESM2_ESM.pdf]

Supporting Information for

Visual features explain dynamic aesthetic experiences across distinct movie content

Mustafa Alperen Ekinci, Nina Buhlmann, Daniel Kaiser

Mustafa Alperen Ekinci
Email: alppekinci@gmail.com

This file includes:
Figures S1 to S6

Supporting Information

Correlation

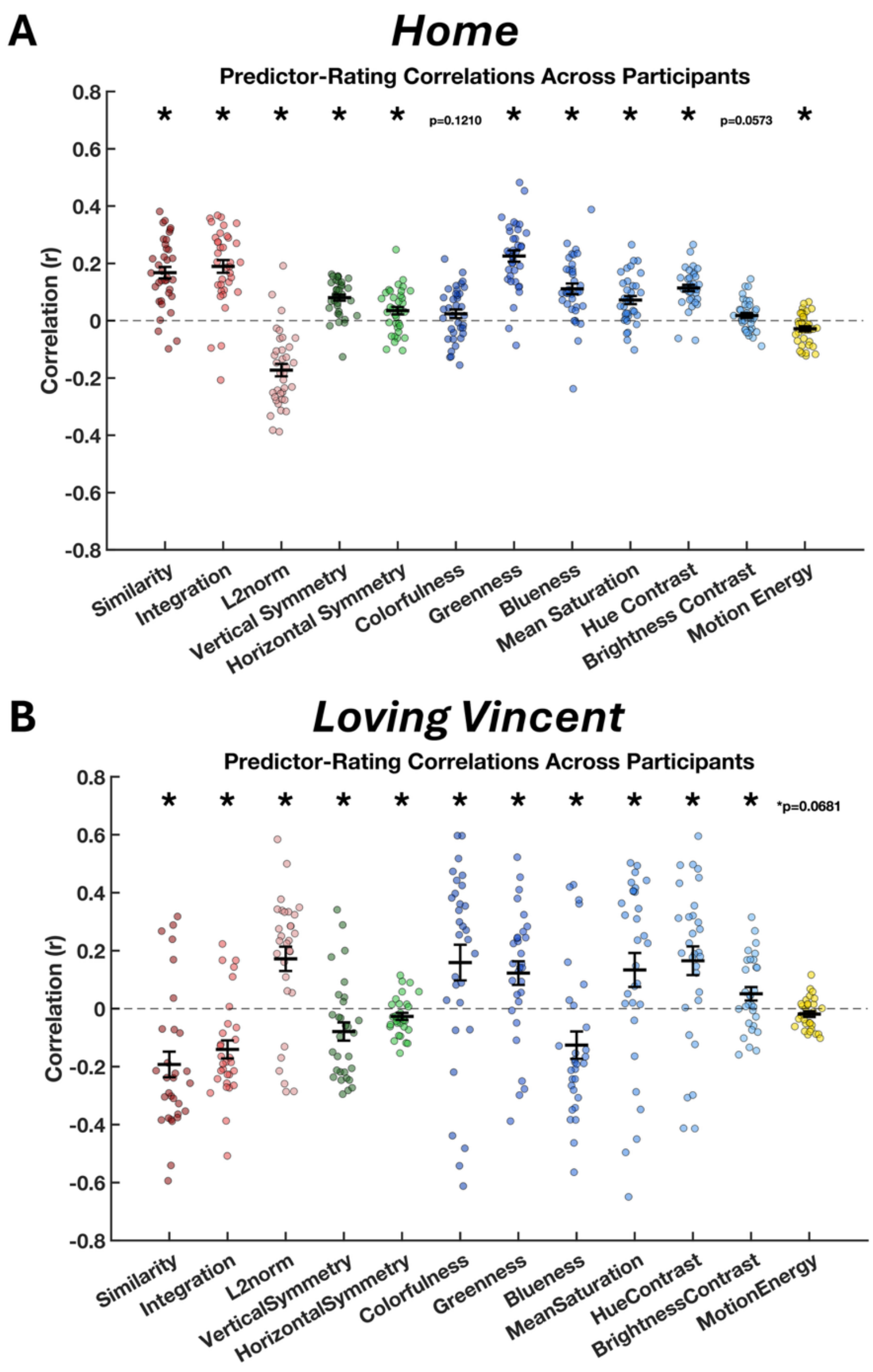

Figure S1. Correlations between individual predictors and aesthetic ratings. In addition to the regression models, we computed correlations between each predictor and the continuous aesthetic ratings. Group-level two-sided one-sample t-tests were performed with FDR correction to assess the relationship between each predictor and dynamic aesthetic experience. Error bars indicate SEM, asterisks indicate $p < 0.05$.

Partial correlation

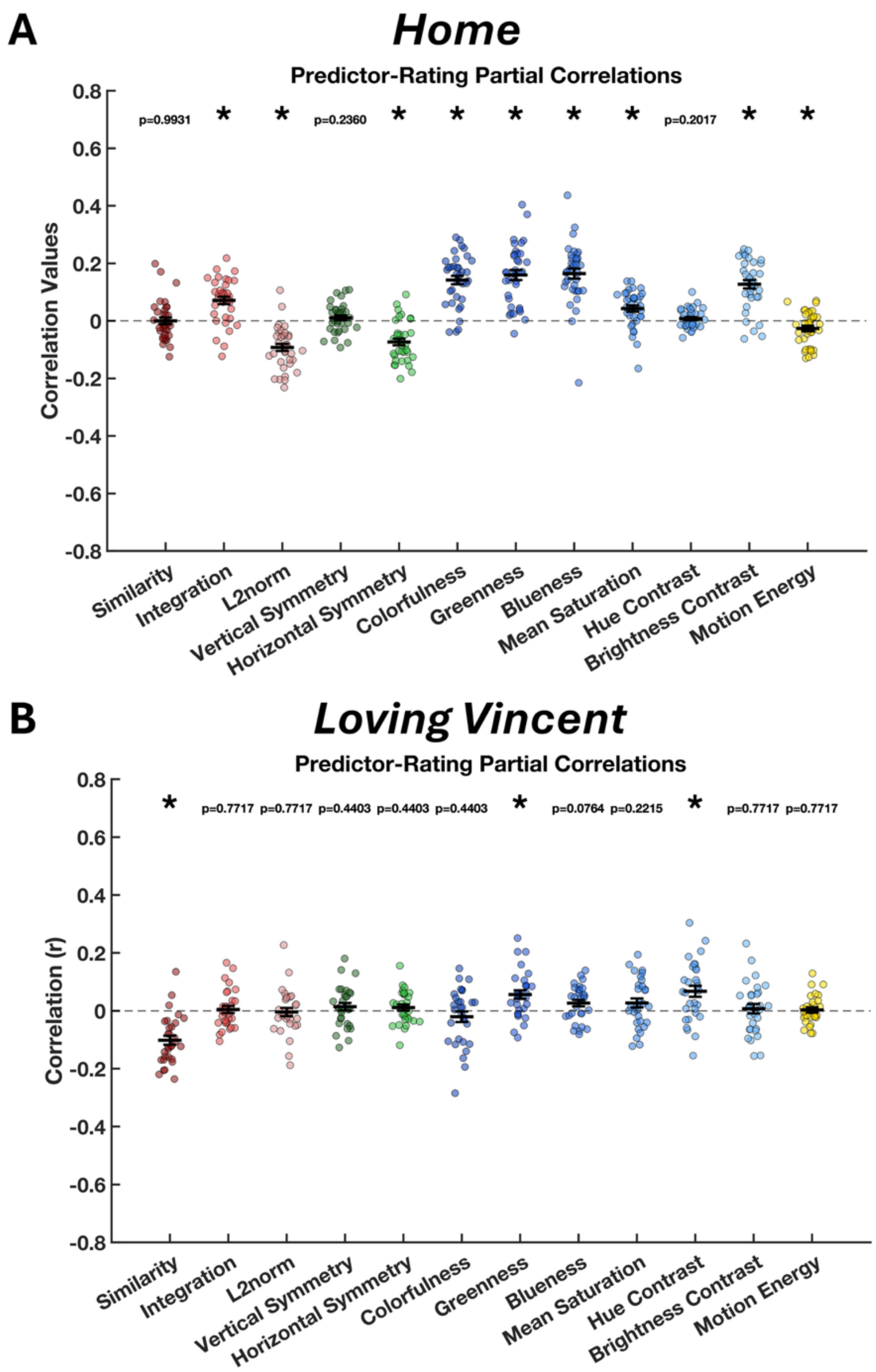

Figure S2. Partial correlations between individual predictors and aesthetic ratings. Partial correlations were computed to examine the relationship between each predictor and aesthetic ratings while controlling for the influence of all other predictors. Group-level two-sided one-sample t-tests were performed with FDR correction to assess the relationship between each predictor and dynamic aesthetic experience. Error bars indicate SEM, asterisks indicate $p < 0.05$.

Beta coefficients

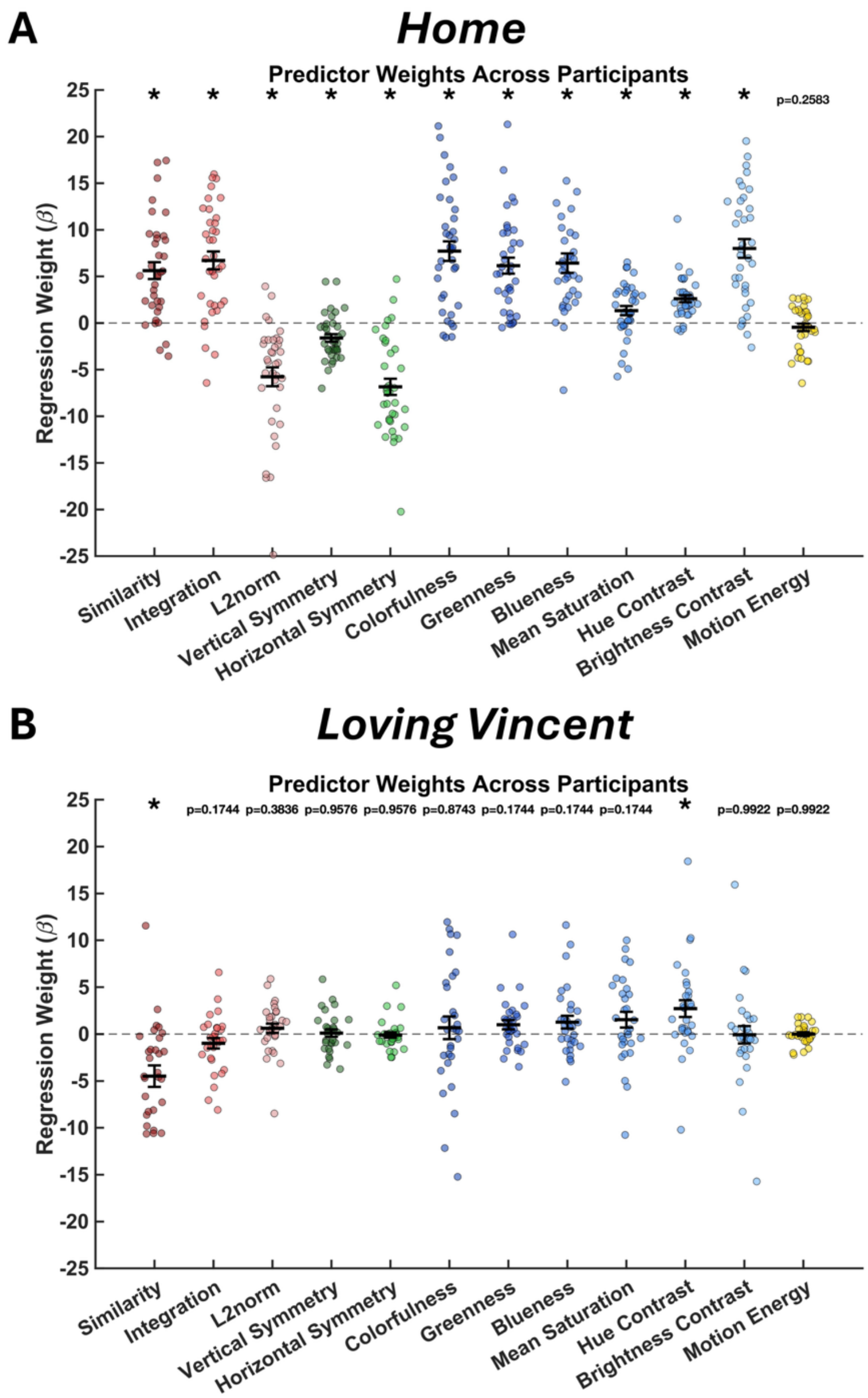

Figure S3. Regression coefficients (beta weights) of predictors from the regression models. These values reflect the unique contribution of each predictor to explaining variance in aesthetic ratings. Group-level two-sided one-sample t-tests were performed on the beta coefficients, with FDR correction applied for multiple comparisons. Error bars indicate SEM, asterisks indicate $p < 0.05$.

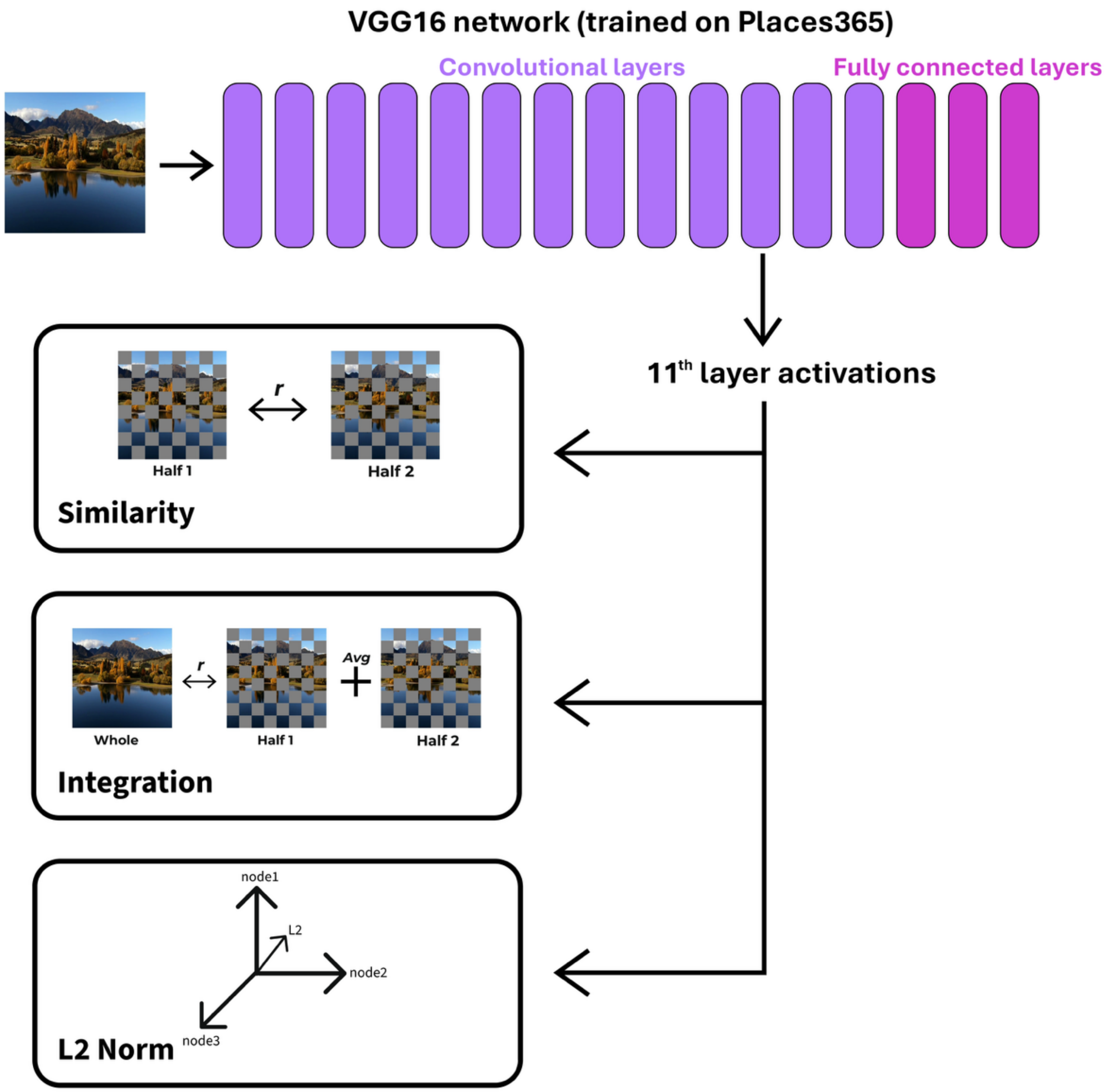

Figure S4. Coding efficiency predictors derived from a deep neural network (VGG16). For each frame, full and two checkerboard-split (8 x 8) image versions were processed, and activations from the 11th layer were extracted. Similarity was computed as the correlation between activations of the two halves, and integration as the correlation between the full image and the average of the two halves (sign inverted so higher values reflect greater integration). The L2 norm was computed from the activation vectors.

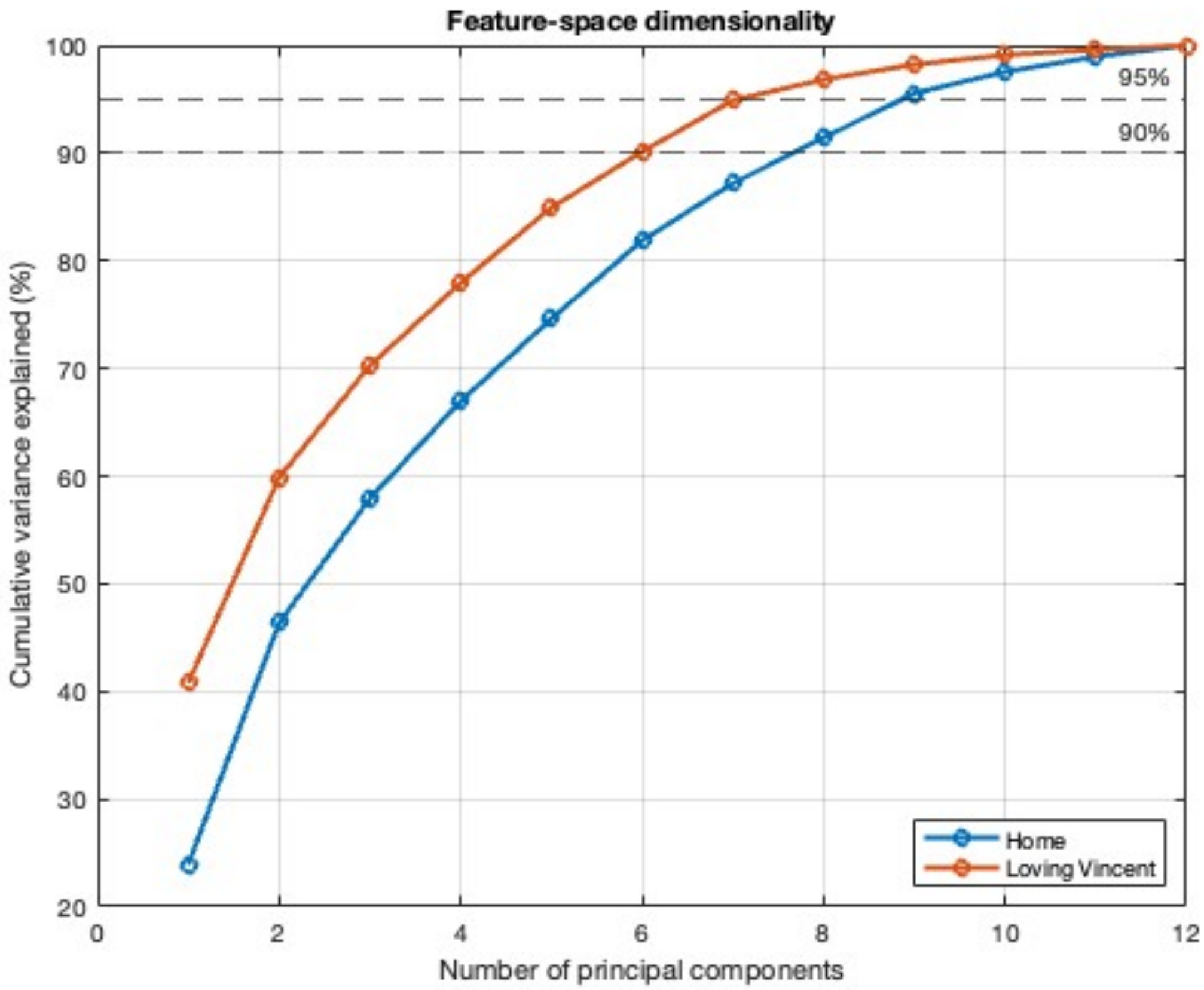

Figure S5. Principal component analysis of the visual feature space. Cumulative variance explained by successive principal components for the 12 visual predictors extracted from *Home* and *Loving Vincent*. *Loving Vincent* exhibited a lower-dimensional feature space than *Home*.

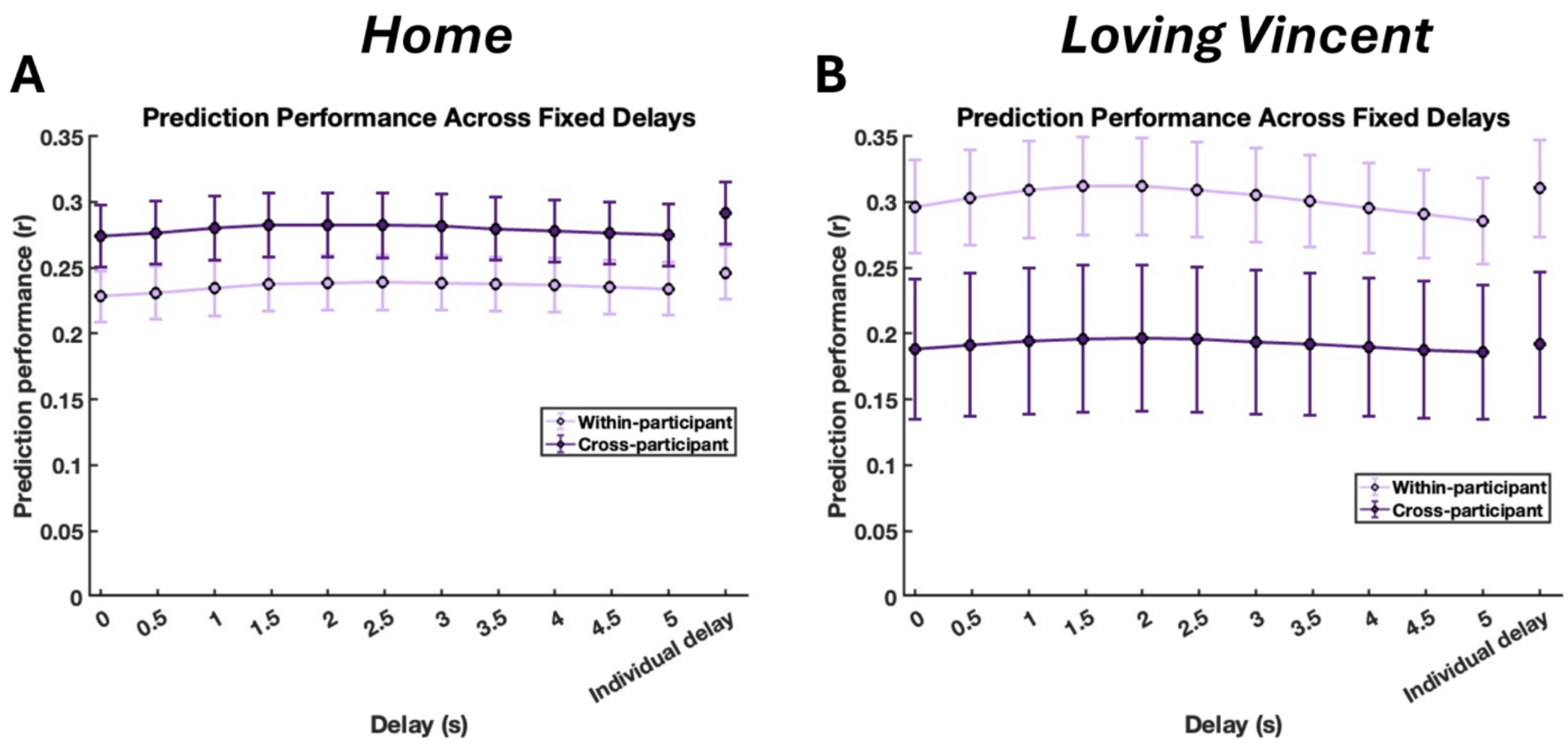

Figure S6. Robustness of prediction performance across fixed response delays. Prediction performance was evaluated after shifting the behavioral ratings by fixed delays ranging from 0 to 5 s in 0.5-s increments. Results are shown separately for *Home* (A) and *Loving Vincent* (B). Prediction performance remained largely stable across the tested delays, while the individually estimated response delays yielded slightly better performance than the fixed-delay approaches.
